# The Emerging Role of the Mitochondrial Respiratory Chain in Skeletal Aging

**DOI:** 10.14336/AD.2023.0924

**Published:** 2024-08-01

**Authors:** Huaqiang Tao, Pengfei Zhu, Wenyu Xia, Miao Chu, Kai Chen, Qiufei Wang, Ye Gu, Xiaomin Lu, Jiaxiang Bai, Dechun Geng

**Affiliations:** ^1^Department of Orthopedics, The First Affiliated Hospital of Soochow University, Jiangsu, China.; ^2^Department of Orthopedics, The First Affiliated Hospital of USTC, Division of Life Sciences and Medicine, University of Science and Technology of China, Anhui, China.; ^3^Department of Orthopedics, Changshu Hospital Affiliated to Soochow University, First People’s Hospital of Changshu City, Jiangsu, China.; ^4^Department of Oncology, Affiliated Haian Hospital of Nantong University, Jiangsu, China.

**Keywords:** bone, mitochondrial respiratory chain, energy, oxidative stress, apoptosis, calcium, mitophagy

## Abstract

Maintenance of mitochondrial homeostasis is crucial for ensuring healthy mitochondria and normal cellular function. This process is primarily responsible for regulating processes that include mitochondrial OXPHOS, which generates ATP, as well as mitochondrial oxidative stress, apoptosis, calcium homeostasis, and mitophagy. Bone mesenchymal stem cells express factors that aid in bone formation and vascular growth. Positive regulation of hematopoietic stem cells in the bone marrow affects the differentiation of osteoclasts. Furthermore, the metabolic regulation of cells that play fundamental roles in various regions of the bone, as well as interactions within the bone microenvironment, actively participates in regulating bone integrity and aging. The maintenance of cellular homeostasis is dependent on the regulation of intracellular organelles, thus understanding the impact of mitochondrial functional changes on overall bone metabolism is crucially important. Recent studies have revealed that mitochondrial homeostasis can lead to morphological and functional abnormalities in senescent cells, particularly in the context of bone diseases. Mitochondrial dysfunction in skeletal diseases results in abnormal metabolism of bone-associated cells and a secondary dysregulated microenvironment within bone tissue. This imbalance in the oxidative system and immune disruption in the bone microenvironment ultimately leads to bone dysplasia. In this review, we examine the latest developments in mitochondrial respiratory chain regulation and its impacts on maintenance of bone health. Specifically, we explored whether enhancing mitochondrial function can reduce the occurrence of bone cell deterioration and improve bone metabolism. These findings offer prospects for developing bone remodeling biology strategies to treat age-related degenerative diseases.

## 1. Introduction

The spine and joints play crucial roles in human movement and weight bearing. The spine serves primarily to protect the bone marrow and support body weight, while the structures of the joints and bones enable the body to move with ease [1-3]. Bone metabolism disorders and degenerative changes encompass osteoporosis, intervertebral disc degeneration, and osteoarthritis (OA) [4-6]. Osteoporosis is a systemic bone disease resulting from an imbalance of osteoblast and osteoclast coupling, leading to the destruction of bone microstructure [7]. Degenerative changes in the spine are primarily caused by abnormalities in nutrient metabolism in the intervertebral discs and matrix metalloproteinase expression, as well as increased levels of inflammatory mediators and apoptosis [8]. Osteojoint degeneration is marked by the wearing down of articular cartilage, the formation of osteophytes, and inflammation of the synovial tissue. This leads to the gradual breakdown of the cartilage and the extracellular matrix that supports it [9]. In the context of joint disorders in elderly individuals, several main causes stand out sharing one common characteristic: correlation with natural aging[10-12]. Additionally, it is worth noting that metabolic disorders affecting functional cells may lead to greater susceptibility to the development of bone diseases [13, 14].

**Figure 1.**
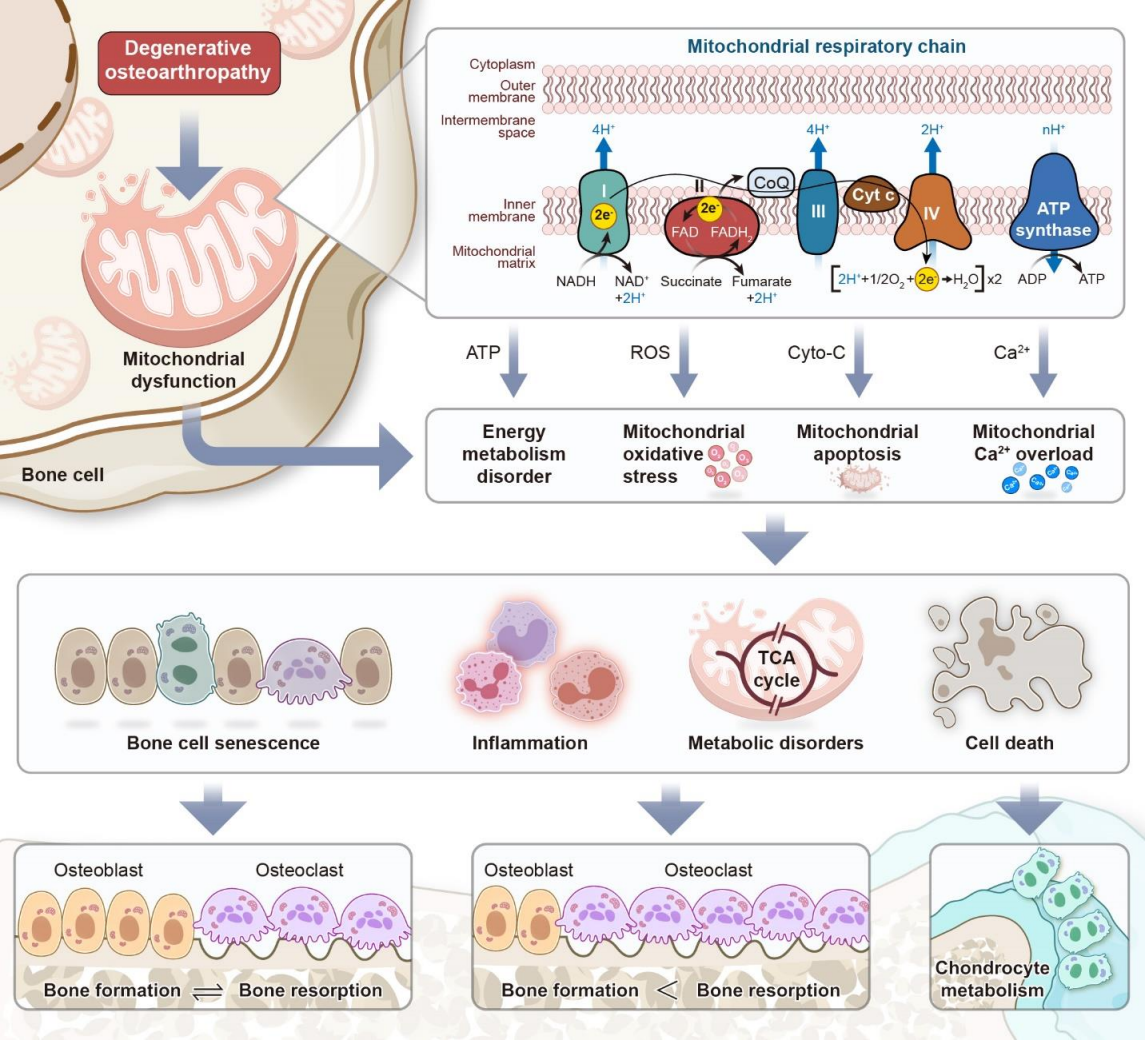
**The MRC in bone homeostasis**. Degenerative osteoarticular diseases are characterized by an imbalance in osteoblast-osteoclast coupling in bone tissue and chondrocyte degeneration. The dysfunction of the MRC within the bone microenvironment may be the cause of this issue, resulting in the blockage of ATP production and leading to the release of ROS and cytochrome c (cyto-c), as well as unbalanced calcium homeostasis. Ultimately, this leads to metabolic disorders in bone-related cells and worsens osteodyscrasia as the body ages.

Bone is a central organ that is vital for maintaining overall body health. It develops through two ossification processes, intramembranous or intrachondral, and undergoes remodeling throughout one's life [15, 16]. The balance of bone remodeling is dependent on the close coordination between bone resorption and bone formation, along with the mutual regulation of different cells within the bone [17-19]. The proper functioning of bones depends on both their structural integrity and the regulation of the bone microenvironment. Local inflammation, oxidative stress, and metabolic immune disorders can disrupt the balance of the bone microenvironment, lead to abnormal bone aging, and ultimately result in bone-related diseases [20-22]. Any factor that disturbs bone aging can negatively affect bone development and lead to malformations. Therefore, the key to regulating the internal metabolic imbalance of various bone diseases lies in coordinating the functional activities of various cell types, thereby providing a healthy, stable internal microenvironment for the growth of bone tissue.

The regulation of intracellular organelles is essential for maintaining cell homeostasis, particularly regarding functions related to bioenergetics, such as energy metabolism and biological regulation [23-25]. As the primary site of aerobic respiration in cells, mitochondria play a crucial role in regulating body homeostasis, generating energy to sustain normal physiological activities [26, 27]. Research has also demonstrated their importance in cell growth, differentiation, information transfer, calcium homeostasis, and metabolic senescence [28-33]. Furthermore, mitochondria control the entry and export of proteins, lipids, and metabolites, while safeguarding the cytoplasm from harmful substances [34, 35]. As individuals age, mitochondrial function naturally declines because of changes in mitochondrial dynamics, active oxygen content, and metabolites [36]. These changes can cause dysfunction in the electron transport chain and oxidative phosphorylation (OXPHOS), leading to the development of age-related diseases [37].

Mitochondrial dysfunction is a hallmark of both cellular senescence and chronic degenerative diseases. Recent studies have revealed that various bone-related cells exhibit morphological and functional abnormalities resulting from disrupted mitochondrial homeostasis [38, 39]. De-energized mitochondria with irregular morphology and function resulting from disrupted mitochondrial homeostasis have also been observed in bone-related pathological conditions [40-42]. Therefore, mitochondrial homeostasis may be involved in the occurrence and progression of cell differentiation in bones. Mitochondrial dysfunction is believed to be the root cause of aging, inflammation, and oxidative stress imbalance [27, 43, 44]. During bone degeneration, the bone microenvironment is disrupted, causing bone-related cells to exhibit abnormal metabolic and biological function behaviors. Additionally, mitochondrial DNA (mtDNA) is subjected to varying degrees of oxidative damage, resulting in impaired cellular energy metabolism, cellular dysfunction, and even cell death [45, 46]. Simultaneously, the continuous presence of low-level inflammation in the body can hinder immune system activation through various mechanisms. This results in the buildup of inflammatory metabolites, which can worsen bone building [47]. Given the central role of the mitochondrial respiratory chain (MRC) in mitochondrial energy production, large numbers mutations in its components have been linked to metabolic or degenerative diseases, predominantly affecting tissues or cells that rely heavily on high levels of adenosine triphosphate (ATP) [48-50]. In this paper, we examine the current understanding of MRC regulation in bone homeostasis. Specifically, we focus on the regulation of energy metabolism, oxidative stress, apoptosis, calcium homeostasis, and mitophagy that impact bone metabolism throughout the body (Fig. 1). Our goal is to determine whether improving mitochondrial function can actively enhance bone metabolism and potentially serve as a treatment for osteoporosis, OA, and other bone degenerative diseases.

## 2. Mitochondrial structure of bone-associated cells

Mitochondria are semiautonomous organelles that have a double-membrane structure. The chemical components of mitochondria mainly comprise water, proteins, and lipids, but they may also include small molecules, such as coenzymes and nucleic acids [51-53]. Mitochondria can be divided into four functional regions: the outer mitochondrial membrane (OMM), the mitochondrial membrane gap, the inner mitochondrial membrane (IMM), and the mitochondrial matrix [54]. The OMM is smooth and serves as the boundary membrane for the organelle [55]. The inward folding of the IMM results in the formation of cristae, which facilitate a great number of biochemical reactions within the MRC, thereby increasing the capacity for ATP production [56]. Notably, the IMM serves as the primary site for ATP synthesis within mitochondria. The mitochondrial membrane gap is situated between the inner and outer layers of the mitochondrial membrane, while the IMM surrounds the mitochondrial matrix [57]. The size of mitochondria varies across different tissues, and is influenced by intracellular metabolism [58].

Mitochondria are morphologically dynamic; however, most are short, with a rod-like shape and granular appearance. Morphology is dependent on both the biological species and the physiological state. In bone tissue, cells are typically categorized into three types: osteoblasts, osteoclasts, and osteocytes [59-61]. Mitochondria in osteoblasts possess a circular bilayer membrane structure and are dispersed throughout the cytoplasm, typically embedded in the rough endoplasmic reticulum (ER) [62, 63]. The IMM is wrinkled, forming a characteristic "crest" that protrudes outward. In osteoblasts, mitochondria serve two functions: removal of Ca^2+^ ions from the cytoplasm and energy processing [64, 65]. Osteoclasts are the only bone cells capable of absorbing mineralized tissues and local inflammatory lesions [66, 67]. Their formation is an energy-intensive process that arises from the fusion of mononuclear macrophages, which differentiate from myeloid progenitors in the bone marrow, and relies on high metabolic activity. The cytoplasm of osteoclasts contains large numbers of mitochondria that provide continuous energy for absorption [68, 69]. The mitochondrial number increases significantly during the differentiation process in human osteoclasts. Additionally, once the osteoclasts have reached maturity, their mitochondrial size also increases, and the cristae become abundant and arranged in a complex tubular network [70, 71]. Osteocytes, which are terminally differentiated cells of the osteoblast lineage, comprise approximately 90% of the total cells in adult bone [72]. Osteocytes have larger mitochondria than osteoblasts, although their number is lower [73]. Research has shown considerable intercellular mitochondrial transfer in osteocytes, which maintains the dynamic regulation of damaged cells and bone homeostasis [74, 75]. The normal survival of chondrocytes is dependent on the structural integrity of their mitochondria. Under physiological conditions, chondrocyte mitochondria are typically oval in shape. However, when mitochondrial damage occurs, irregularly shaped mitochondria become more prevalent, and the density of mitochondria decreases. Additionally, folded cristae are lost, and the mitochondrial membrane is damaged. As OA progresses, the swelling of chondrocyte mitochondria becomes increasingly apparent [76-78].

## 3. Regulation of the MRC in bone metabolism

**Figure 2.**
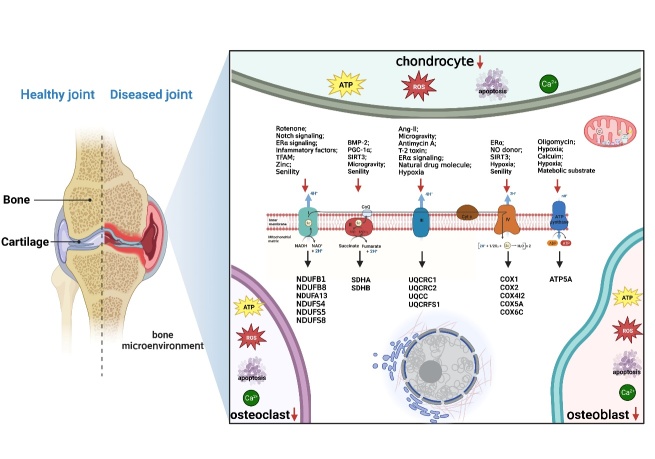
**Structure and exogenous regulatory factors of the MRC**. The MRC provides 95% of the energy required for cell survival, which is achieved by the activities of five mitochondrial respiratory enzyme complexes located in the IMM. These protein complexes carry out a series of tasks involving electron transfer and proton pumping. Mitochondrial respiratory complexes I-V contain specialized functional subunits that play a crucial role in regulating bone metabolism and differentiation. Additionally, certain chemical drugs and exogenous signaling proteins modulate mitochondrial complex activity, which can impact electron transport chain functionality and cellular processes. Disrupted MRC transmission can lead to mitochondrial homeostasis disruption, which may result in undesirable bone phenotypes. This dysfunction is the primary cause of bone aging and metabolic imbalance. Disorder within the bone microenvironment can lead to abnormal metabolism and functioning of bone-related cells, resulting in impaired energy metabolism, redox instability, cellular dysfunction, and cell death.

Mitochondria are responsible for producing energy via oxidization, and the majority of the energy required for cell survival is generated by the MRC [79, 80]. This chain is primarily facilitated by a group of mitochondrial respiratory enzymes comprising five complexes located within the IMM [81, 82] (Fig. 2). These protein complexes work together to transfer electrons and pump out protons within the inner membrane. A significant cause of bone disorders is the abnormal metabolism of the MRC enzyme complex [83-85].

Respiratory chain complex I (CI), also referred to as nicotinamide adenine dinucleotide (NADH)-Q reductase, is the largest membrane complex responsible for transferring the electrons of NADH to coenzyme Q (coQ) [86, 87]. CI is responsible for initiating and creating the electrochemical gradient necessary for ATP synthesis. A previous study proposed that osteoporosis may be linked to the build-up of pathogenic mtDNA mutations that occur with age [85]. These mutations can result in respiratory chain defects, ultimately leading to notable impairments in osteoblast CI with age. A recent mass spectrometry imaging study of osteoblasts in osteoarthritic imaging patients revealed that 60% of the volunteers had defects in NADH:ubiquinone Oxidoreductase Subunit B8 (NDUFB8), 40% also had defects in genes associated with NDUFA13. Both NDUFB8 and NDUFA13 are CI modules and exhibit a strong correlation in their function [88]. Long used a broad-spectrum insecticide, rotenone, an active substance extracted and isolated from rattan plants, was shown to be a respiratory inhibitor that primarily targets CI [89, 90]. Specifically, rotenone acting on a component between dehydrogenase and coQ leads to inhibited cell activity and hindered ATP synthesis [91]. Studies have demonstrated that epigallocatechin gallate and resveratrol can enhance mitochondrial parameters, such as basic and maximum respiration, reserve respiration capacity, and ATP production, ultimately leading to the differentiation of human fetal osteoblasts via the pAMPK-AdipoR1-PGC1α pathway. Rotenone counteracts the osteogenic effects of these two dietary polyphenols [92]. A study by Porwal *et al.* found that guava triterpene-enriched extract increased glycolysis and mitochondrial respiration *in vitro*, leading to induced osteogenic differentiation through the activation of GSK-3β phosphorylation-mediated Wnt/β-catenin pathways. Interestingly, the inhibition of osteoblast differentiation was only observed with rotenone, and not with 2-deoxyglucose, which is commonly used to block glycolysis [93]. These results suggest that CI-mediated OXPHOS and ATP generation may play critical roles in osteogenic differentiation. The Notch signaling pathway participates in regulation of cell fate decisions and maintaining homeostasis in adult tissues during mammalian development. Research has demonstrated that Notch activation inhibits bone mesenchymal stem cell (BMSC) glycolysis and transcription of MRC genes, specifically the CI genes NDUFC1, NDUFS5, NDUFAF2, and NDUFAF4, through AMPK signaling cascades. Consequently, Notch signaling inhibits osteoblast differentiation and helps to maintain bone marrow mesenchymal progenitor cells after birth [94]. Rothmund-Thomson syndrome (RTS), a genetic disorder that is inherited in an autosomal recessive pattern, is characterized by symptoms including short stature, skeletal abnormalities, and an increased risk of developing osteosarcoma. Individuals with RTS exhibit reduced bone morphogenesis and have abnormalities in mitochondrial respiratory CI genes, especially NDUFA7, NDUFB1, NDUFB2, and NDUFS8 as well as large chromosomal deletions including genes involved in the bone development pathway of pre-osteoblasts and osteoblasts. Nevertheless, studies have shown that higher levels of accessory and catalytic subunits in mitochondrial CI are associated with the development of RTS, indicating that increased expression of genes that produce ATP in mitochondria may contribute to the development of osteosarcoma [84].

Fully differentiated osteoclasts are functional cells that require large amounts of ATP to complete bone resorption [95]. These cells have higher levels of electron transportases, increased expression of MRC proteins, and higher rates of mitochondrial oxygen consumption [96, 97]. Levels of the MRC protein CI subunit NDUFB8 in osteoclasts of aging mice were found to be lower than those of young, wild-type mice [85]. Kim's team discovered that, in postmenopausal estrogen deficiency-induced osteoporosis, estrogen signaling mediated by estrogen receptor alpha (ERα) reduces the activity of OXPHOS and CI subunit gene expression in osteoclast progenitors, inhibiting osteoclast differentiation by reducing ATP production. They also found that rotenone has the ability to promote the apoptosis of osteoclast progenitors through Bak/Bax, ultimately leading to reduced osteoclasts [97]. Interestingly, rotenone can also effectively treat inflammatory bone loss by inhibiting osteoclast differentiation through the c-fos/NFATc1 pathway in a dose-dependent manner [98]. However, some researchers have shown that, while rotenone enhances osteoclast activity at cytotoxic doses, enzymes associated with the glycolytic pathway are also found near the actin rings of polarized osteoclasts. This localization suggests that energy-demanding activities associated with bone degradation occur in this area [70]. The variation in output may be attributed to the different substrate media used to induce the osteoclastic differentiation process. NDUFS4 encodes a nuclear-encoded subunit accessory of CI. Jin *et al.* utilized an NDUFS4 deletion as a model for mitochondrial dysfunction, demonstrating that regular mitochondrial metabolism prevents proinflammatory macrophage activation and acts as a rheostat of innate immunity. Furthermore, mice with global NDUFS4 deletion exhibit an increased bone mass phenotype, in which histological staining indicated a reduction in both the quantity and size of osteoclasts, while osteoblasts remained unchanged. Further investigation involved extracting bone marrow macrophages (BMMs) from NDUFS4 knockout mice, which were then cultured *in vitro* with induction of receptor activator of nuclear factor-κB (RANKL). The results indicated decreases in the number and volume of osteoclasts and bone resorption activity, accompanied by enhanced macrophage activation and inflammation. Additionally, Toll-like receptor 4/2 signaling was identified as an important mediator in this process [99].

Cartilage serves as an attachment to the surface of joints, providing support, cushioning, and lubrication for joint movement [100]. Mature chondrocytes are found within the interstitial cartilage. The decreases in basic oxygen consumption and intracellular ATP levels of chondrocytes in OA are thought to be due to reduced mitochondrial biogenesis and impaired mitochondrial function [101]. A study demonstrated a statistically significant trend indicating reduced CI activity in chondrocytes from older patients compared with normal human chondrocytes. CI activity decreases in response to proinflammatory factors, such as the cytokines interleukin (IL)-1β and tumor necrosis factor (TNF)-α, which in turn leads to decreases in proteoglycan levels and cartilage function [102]. Furthermore, decreased expression of mitochondrial transcription factor A (TFAM) may be responsible for the inhibition of chondrocyte mitochondrial CI activity in OA. Pharmacologically activating AMPK signaling was shown to improve the critical viability of chondrocytes by increasing the expression of complexes (e.g., CI) and the level of ATP in chondrocytes through TFAM-mediated activation of the silent mating type information regulation 2 homolog (SIRT)1/PGC-1a pathway [103]. In a previous study, researchers had discovered that the progression of OA was mimicked in chondrocytes treated with monosodium iodoacetate (MIA). This treatment reduced the expression of several mitochondrial complex subunits, including NDUFB8 of CI, SDHB of CII, UQCRC2 of CIII, COX2 of CIV, and ATP5A of CV [104]. The negative effects of MIA treatment were ameliorated by zinc treatment, which promoted ATP production and the OXPHOS pathway in chondrocytes. It is evident that maintaining the normal structure and function of CI is crucial for the survival of chondrocytes and to impede the progression of bone and joint degeneration.

**Figure 3.**
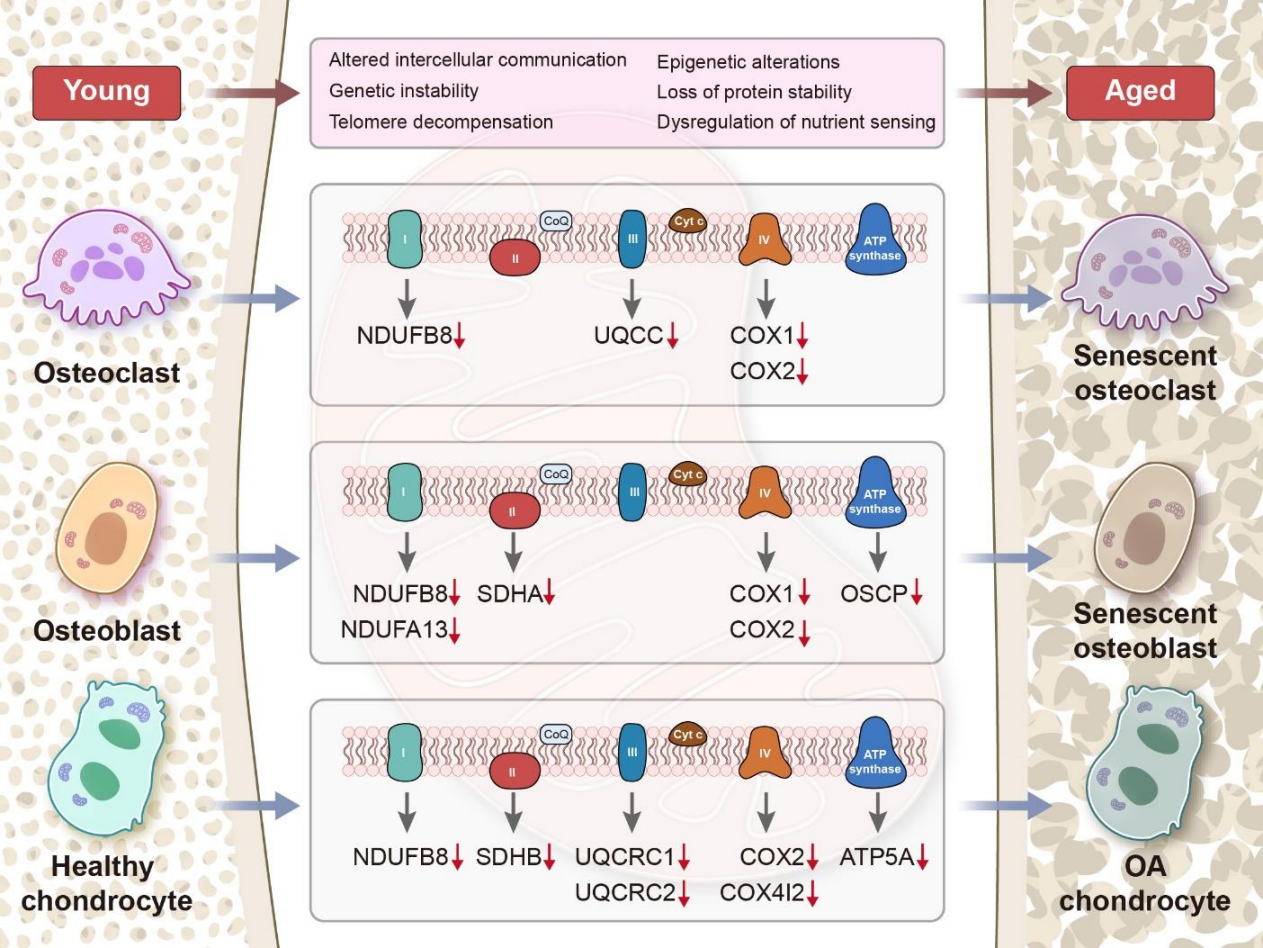
**Age-related changes in MRC subunit activity in bone-related cells**. The activities of osteoblasts, osteoclasts, and chondrocytes are significantly decreased during aging. This decrease is accompanied by downregulation of MRC subunit activity in bone-related cells due to the intrusion of various abnormal metabolic factors or harmful substances in the body. This obstruction of MRC affects cellular energy metabolism and intracellular redox signaling, ultimately accelerating pathological bone deterioration.

Mitochondrial complex II (CII), also known as succinate dehydrogenase-ubiquinone oxidoreductase, does not pump protons; instead, it contributes to the reduction of ubiquinone, which then transfers electrons to complex III (CIII) [105, 106]. Mutations in *SDHA* of CII have been identified in osteoblasts from patients with aging-related osteoporosis, indicating its potential involvement in the disease pathogenesis [88] (Figure 3). *In vitro*, induction of osteoblast differentiation gradually enhanced the activities of mitochondrial CI and CII. The osteogenic regeneration potential of bone morphogenetic protein (BMP) was found to accelerate the adhesion and proliferation of osteoblasts by enhancing the activities of CI and CII. Peroxisome proliferator-activated receptor gamma coactivator (PGC)-1α is an essential regulator of mitochondrial production, responsible for activating the expression of numerous transcription factors involved in mitochondrial components and respiration through transcriptional costimulation. Studies have demonstrated that BMP-2 regulates osteogenic MRC activity through the regulation of PGC-1α [107]. Normal mitochondrial function is essential for physiological osteogenic differentiation. Silent mating type information regulation 2 homolog 3 (SIRT3) is an important mitochondrial deacetylase that participates in the activity of osteoblast CI-V and mitochondrial membrane potential (ΔΨ), leading to alterations in mitochondrial ultrastructure. SIRT3 inhibition was observed to hinder the oxygen-depleting activity of osteoblasts via PGC-1α-SOD2-mediated regulation of mitochondrial function, as evaluated by measurement of CII-driven respiration [108]. The development of space technology has led to the emergence of a new field: microgravity research. A recent study revealed that the stress induced by exposure to microgravity decreases mitochondrial protein and respiratory chain efficiency in human primary osteoblasts. This decrease may be attributed to alterations in CII and interruption of the Krebs cycle, obstructing ATP synthesis and ultimately resulting in impaired osteoblast function [109]. Collectively, these findings suggest that mitochondrial CII is crucial for osteoblast differentiation, which in turn may affect the OXPHOS pathway and energy metabolism in the course of cellular fate regulation.

OA progression is characterized by decreased numbers of chondrocytes, loss of extracellular matrix, and pathological matrix calcification. Further analysis of MRC activity in OA chondrocytes revealed a significant reduction in CII compared with normal chondrocytes. This malfunction of CII disrupts the electron transfer pathway, resulting in reduced ATP production in the mitochondria [110, 111]. Kashin-Beck disease (KBD) is a form of degenerative OA that is endemic to certain regions and is characterized by the degradation of extracellular matrix and necrosis of chondrocytes. A study revealed significant reductions in the activities of CII, CIII, CIV, and CV in KBD chondrocytes compared with those in normal controls. These reductions led to decreases in intracellular ATP content and ΔΨ. Furthermore, KBD cells exhibited release of the proapoptotic factor cyto-c from the intermembrane space and activation of cysteinyl aspartate-specific proteinase (caspase)-9 and 3, resulting in larger numbers of apoptotic chondrocytes [112].

CIII, also known as ubiquinone-cyto-c oxidoreductase, plays a crucial role in the transfer of electrons from QH2 to cyto-c [113]. Angiotensin II, a hormone that regulates water and salt metabolism and blood pressure, has been shown to inhibit the activities of CI, CIII, CIV and promote the apoptosis of calvarial osteoblasts in mice via the c-Jun N-terminal kinase pathway in mice [114]. Simulated microgravity experiments in human primary osteoblasts led to a 60% increase in mitochondrial CIII (including subunits UQCRC1 and UQCRFS1) and a 14% decrease in CIV (including subunits COX5A and COX6C). This resulted in a defect in osteoblast function, which may be due to the impact of microgravity on mitochondrial energy potential and cell status [109]. Additionally, the primary focus of estrogen's effect is on osteoclast progenitor cells, and an increase in the UQCC of the CIII subunit was observed in ERα-deficient BMMs [97]. Antimycin A, an antibiotic isolated from Streptomyces, inhibits electron transfer in reductase, stimulates oxidative stress, and induces the release of cyto-c in mitochondria in osteoblasts. This results in the death of MC3T3-E1 cells together with the loss of intracellular ATP and mitochondrial ΔΨ [115]. Therefore, it can be concluded that antimycin A has a significant effect on mitochondrial function in MC3T3-E1 osteoblasts. In continued research, Professor Choi’s team discovered that several natural molecules, including paeoniflorin, honokiol, and liquiritigenin, offer protection against antimycin A-mediated inhibition of the osteoblast respiratory chain [116-118]. One common effect of these substances is their ability to reduce the dissipation of mitochondrial ΔΨ and loss of ATP. Additionally, they can inhibit the production of superoxide in osteoblast mitochondria. Conversely, antimycin A has been found to delay osteoclast formation by inhibiting ATP production. Regarding the metabolic coupling of osteoblasts and osteoclasts, antimycin A has been shown to promote the release of resorptive mediators associated with osteoclastic differentiation in osteoblasts [119].

Knee cartilage staining of aged mice with OA showed a significant decrease in the expression of subunit UQCRC1 of CIII *in situ*. Additionally, TFAM expression in human chondrocytes, especially that clustered in OA cartilage, was found to be significantly correlated with UQCRC1, suggesting that CIII was involved in cartilage metabolism [103]. T-2 toxin, a mycotoxin produced by Fusarium, has been identified as a possible causative factor of KBD. Research has shown that a concentration gradient of T-2 toxin can reduce the activity of chondrocyte mitochondrial CIII, CIV, and CV, while inducing the intracellular accumulation of reactive oxygen species (ROS). This ultimately leads to the release of cyto-c and activation of caspase-9 and 3 in chondrocytes, which mediate mitochondrial pathway apoptosis [120]. The oxygen level in the environment surrounding articular cartilage stroma and chondrocytes is typically low and can be reduced to very low levels in joint diseases, such as chronic inflammatory arthritis [121, 122]. Studies conducted *in vitro* have demonstrated that the intracellular pH levels of articular chondrocytes exposed to hypoxia become more acidic due to the inhibition of Na^+^/H^+^ exchanger activity. CIII has been identified as a potential site for ROS production. During hypoxia, the depolarization of the mitochondrial ΔΨ may decrease ROS levels, which in turn can mediate the pathophysiological outcome of OA [120]. Additionally, antimycin A-induced inhibition of CIII activity in human articular chondrocytes leads to the synthesis of ROS and the production of other proinflammatory stimuli, such as ILs, prostaglandin E2, and various matrix metalloproteinases (MMPs) [123, 124].

CIV, also known as cyto-c oxidase, is a multisubunit protein complex that facilitates the transfer of electrons from the respiratory chain to oxygen molecules via cytochromes [125, 126]. As humans age, mitochondrial mutations and respiratory chain defects tend to accumulate. However, studies suggest that changes in CIV are not detectable in patients under 35 years of age, and osteoblast assays in older patients do not show significant levels of CIV deficiency. This could be attributed to the limited number of CIV genes in mtDNA [88]. CIV comprises 13 subunits, of which the mtDNA-encoded COX1 and COX2 are crucially important, facilitating the transfer of electrons from cyto-c to oxygen. In osteoblasts, estradiol triggers the expression of the mitochondrial COX1 and COX2, leading to the activation of ERα and subsequent ATP synthesis. However, pretreatment of rat calvarial osteoblasts with methylpiperidinopyrazole inhibits the expression of COX1 and COX2 mRNA by mediating estrogen-induced ERα translocation [127]. Reducing the translocation of ERα from the cytoplasm to the mitochondria ultimately impedes the maturation of osteoblasts. COX1 expression is diminished with age in osteoblasts and osteoclasts in wild-type mice, and this reduction is further exacerbated in PolgA^mut/mut^ mitochondrial ‘mutator’ mice. These observations imply that CIV may be actively involved in bone metabolism, particularly in relation to aging and mitochondrial damage [85].

Mitochondrial dysfunction in chondrocytes with OA may occur because of somatic mutations in mtDNA or in direct response to the effects of pro-inflammatory mediators, such as cytokines, prostaglandins, and nitric oxide (NO). The inflammatory environment induced by NO causes chondrocytes to undergo apoptosis and depolarization of ΔΨ [128, 129]. Previous studies that used sodium nitroprusside as an NO donor to stimulate normal chondrocytes found that the activity of CIV was significantly lower than that of control cells. This was accompanied by increased mRNA levels of caspase-3 and 7 as well as downregulation of BCL-2 protein, thereby leading to matrix loss and cartilage mineralization [129]. Treatment of chondrocytes with NOC-12, another NO donor, resulted in reductions in the activities of CI, CIII, and CIV, and an increase in mitochondrial mass. These reductions in mitochondrial complex activity lead to dysfunction in the electron transfer pathway, ultimately causing chondrocytes to increase anaerobic metabolism to avoid overproduction of ROS [130]. Immunofluorescence staining of COX in frozen sections of mouse femurs showed that COX staining was limited to the lateral growth plate and periosteum, which are adjacent to blood vessels, and was virtually undetectable in the central chondrocytes. In 13-day-old mice, COX staining was primarily detected in proliferating chondrocytes after the formation of secondary ossification centers. In 1-month-old mice, the majority of cells in the growth plates were COX-positive, with the highest level of staining observed in proliferating chondrocytes [131]. These findings suggest that chondrometabolism is partially aerobic and that respiration is only activated later in postnatal development, when blood vessels surround the growth plate to provide sufficient oxygen and energy levels. In recent years, studies have demonstrated that hypoxia leads to decreased mitochondrial ΔΨ and activity of CIV and inhibits cyclic AMP response element-binding protein (CREB) phosphorylation in human cartilage C28/I2 cells. However, the protein RhoA has been found to inhibit the hypoxia-induced reduction in CREB phosphorylation and induction of apoptosis, while simultaneously improving mitochondrial function [132]. SIRT3 is a deacetylase located in mitochondria. One of its targets is CIV subunit 4 isoform 2 (COX4I2), a subtype of the COX4 subunit, which plays a crucial role in maintaining cartilage integrity. Studies have shown that SIRT3 regulates COX4I2 in a deacetyl-dependent manner. Furthermore, SIRT3 deficiency and mitochondrial respiratory dysfunction have been found to accelerate the destruction of cartilage extracellular matrix and the progression of OA [133].

The final component of the MRC, CV or ATP synthase, is made up of two functional protein complexes: hydrophilic F0 and hydrophobic F1 [134, 135]. Its primary function is to transfer protons through the electrochemical gradient in mitochondria, ultimately providing cellular energy via synthesis of ATP from the raw materials ADP, Pi, and Mg^2+^ [136]. CV is a crucially important enzyme in the production of aerobic energy in the postmitochondrial electron transport train. Its regulatory effects are dependent on specific cellular metabolic conditions, including hypoxia, metabolic uncoupling of mitochondria and calcium concentration [137]. A deficiency of CV subunit OSCP was identified in the osteoblasts of elderly patients [88]. In mammalian cells, oligomycin hinders the process of OXPHOS in the CV of the MRC by binding to F0, altering the configuration of the enzyme [138]. This, in turn, inhibits proton flow in the mitochondrial membrane gap, preventing it from returning to the mitochondrial matrix. Consequently, ATP synthesis is blocked, leading to a shortage of energy required for biological metabolism. Chuang and colleagues discovered that oligomycin administration resulted in a substantial decrease in simvastatin-mediated stimulation of both ATP content and osteoblast proliferation. Additionally, upregulation of the cell cycle proteins D2 and BCL-2 was completely reversed by oligomycin treatment [139]. Christian *et al*. discovered that transferring mitochondria from donor bone marrow stromal cells to the same batch of recipient bone marrow stromal cells, which had been passaged, resulted in improved BMSCs proliferation and migration capacity [140]. Additionally, this transfer led to an increased osteogenic capacity, which was attributed to an enhancement in aerobic metabolism. Transplantation of modified BMSCs into a rat cranial bone defect model led to an increase in bone formation at the site. However, when the rats were treated with oligomycin, their OXPHOS activity and ATP production decreased, which ultimately downregulated bone formation. In osteoclasts, differentiation has been linked to the transfer of metabolic substrates. Studies have revealed that the use of oligomycin can impede ATP production, thereby slowing down the process of osteoclast differentiation [141].

Chondrocyte metabolism is closely linked to CV health. Research indicates that the use of antimycin A can disturb the proton gradient and ATP synthesis, resulting in high levels of superoxide production and a marked decrease in mitochondrial coupling efficiency. This can have serious consequences for the respiratory ability of chondrocytes, ultimately leading to apoptosis [142]. MRC dysfunction has been found to trigger inflammatory responses in chondrocytes and may also regulate extracellular matrix remodeling of chondrocytes. When chondrocytes were treated with oligomycin, the mRNA levels of the MMP family, specifically MMP-1 and MMP-3, were upregulated, leading to decreases in proteoglycan levels and cartilage function [124]. The activation of PKC-βI, induced by the production of endogenous ROS, is believed to cause chondrocyte death. Furthermore, the presence of oligomycin significantly enhanced cell survival against poly-_L_-lysine, indicating that ROS production may occur through CV in the mitochondrial MRC [143]. Table 1 lists the functional subunits of the MRC complexes that are altered in degenerative joint diseases, along with their potential roles in bone physiology and metabolism. We found declining activity of the MRC complexes and their functional subunits in bone-associated cells during skeletal aging. This may be attributable to internal mitochondrial dysregulation caused by the accumulation of metabolic waste and activation of damage-related signals due to aging.

## 4. The MRC and energy regulation in bone metabolism

Bone is a remarkable organ that undergoes constant remodeling through the regulation of bone formation and resorption. Cellular metabolism, which provides energy for all cellular activities, also plays an important role in regulating cellular behavior. Efficient energy metabolism and regulatory programs are closely involved in both skeletal development and maintenance of homeostasis [144, 145]. Additionally, the energy-generating substrates involved in metabolism exhibit varying efficiencies during different stages of cellular differentiation [146]. The mitochondrial OXPHOS system is crucial for cell metabolism, comprising five enzyme complexes and two mobile electron carriers that function in the MRC. The production of ATP is facilitated by coupling the oxidation of reducing equivalents, along with the generation and subsequent dissipation of proton gradients in the IMM. Osteoblasts and osteoclasts, the primary cells responsible for bone formation and resorption, require significant amounts of energy to carry out their biological functions [147, 148]. While early chondrocytes predominantly utilize anaerobic glycolysis for energy, they also rely on intact mitochondria and mitochondrial respiration in later stages [9]. Abnormalities in mitochondrial respiratory coupling of bone-associated cells in the skeleton and impairment of energy metabolism can lead to a disruption of bone metabolic homeostasis and the development of bone metabolic diseases.

Osteoblasts mature and attach to the surface of bones. Some become encapsulated in the bone matrix as osteoblasts or become bone lining cells, while others undergo apoptosis and are metabolized by the body. Bone marrow is a relatively hypoxic environment, especially in ossification centers. Throughout the growth, development, and continuous remodeling of bone, osteoblasts play a critical role in producing new bone mass and increasing bone mass. These processes require significant amounts of energy [149]. Mitochondria consume over 90% of an organism's oxygen molecules. As the terminal electron acceptor of the MRC, oxygen plays a crucial role in the OXPHOS of ATP, which is essential for sustaining life through energy metabolism. Indeed, MRC-dependent OXPHOS generate more ATP than glycolysis. However, recent research has shown that, even though mature osteoblasts have more mitochondria, they tend to utilize aerobic glycolysis to convert glucose into lactate, even in the presence of sufficient oxygen. Further research has shown that the aerobic glycolytic reaction of glucose is responsible for generating approximately 80% of the energy in osteoblasts. As osteoblasts mature, the contribution of mitochondrial respiratory reactions to energy production gradually decreases, with aerobic glycolysis becoming the primary mode of energy acquisition [147, 150]. In another study, researchers observed that, during osteogenic differentiation of human mesenchymal stem cells (hMSCs), there was an increase in mitochondrial OXPHOS, but no significant change in glycolytic levels compared with undifferentiated cells. In contrast, Chen *et al*. found that undifferentiated hMSCs had higher levels of glycolytic enzymes and lactate [151]. These findings suggest that cells of different origins may have distinct metabolic patterns during osteogenic differentiation. Although researchers can simulate microenvironmental conditions of differentiation to some extent *in vitro*, they cannot replicate the compensatory effects between nutrients, or the ratios of nutrients utilized by cells at different stages of differentiation. Consequently, scientists are actively working to overcome these limitations. Initial investigations have shown that interference of the oxidative MRC of MSCs via exogenous substances or pharmacological inhibition can lead to changes in osteoblast function and ultimately result in decreased osteogenic capacity.

Osteoclasts originate from primitive mononuclear progenitor cells and undergo fusion to form multinucleated cells. This process necessitates metabolic reprogramming to maintain biosynthetic substrates. Osteoclasts, which are responsible for bone resorption, require significant amounts of ATP produced through glycolysis and OXPHOS [68]. They play a crucial role in bone remodeling, either by promoting their own migration through rearrangement of the F-actin and microtubule cytoskeleton or by sustaining their survival and facilitating bone resorption. To accomplish these tasks, osteoclasts require significant amounts of ATP, which they release from their mitochondria and store in the cytoplasm [95]. Mature osteoclasts exhibit augmented mitochondrial protein content and decreased intracellular ATP levels. However, the scarcity of intracellular ATP results in irregularities in mitochondrial cristae, enhancing their phagocytic activity. Unlike the osteogenic differentiation of BMSCs, the differentiation of macrophages to osteoclastic precursor cells in response to RANKL stimulation involves significant increases in both MRC-dependent OXPHOS and aerobic glycolysis. The upregulation of MRC complexes and abundant intracellular ATP levels in fully differentiated osteoclasts indicate that mature osteoclasts primarily rely on OXPHOS for energy production to facilitate the biosynthesis of essential components required for differentiation [152]. Previous studies have demonstrated that inhibiting OXPHOS can hinder the differentiation of osteoclast precursor cells into mature osteoclasts. *In vivo* knockdown (KD) of NDUFS4 has been shown to effectively impede osteoclast differentiation, ultimately resulting in osteosclerosis [99]. TFAM, a transcription factor that specifically enhances mtDNA transcription in the presence of mitochondrial RNA polymerase and transcription factor B, is important for maintaining healthy mitochondrial genomes. KD of mitochondrial TFAM in osteoclasts using the cathepsin K recombinant mouse model resulted in significantly reduced intracellular ATP levels and promotion of apoptosis [153]. Furthermore, the *in viv*o KD of Ldha or Ldhb, which encode catalytic enzymes important for aerobic glycolysis, resulted in reductions in both glycolysis and OXPHOS that led to impaired osteoclast formation [154]. These findings indicate that mitochondrial respiration and glycolysis are interdependent during osteoclast differentiation, and that their combined effects influence the differentiation process and eventual maturation of osteoclasts. Under RANKL-induced differentiation of osteoclasts, mitochondrial respiration is enhanced. However, blocking ATP production with mitochondrial complex inhibitors, such as rotenone and antimycin A, or ATP synthase inhibitors, such as oligomycin, impedes osteoclast formation. The transcription factor MYC has been shown to induce the expression of both estrogen-related receptor α signaling and electron transfer chain genes, which are critical for activating OXPHOS. Interestingly, mice with an osteoclast-specific knockout of MYC exhibit increased bone mass and are effectively protected against bone loss induced by oophorectomy [155]. However, research on osteoclasts that exhibit the metabolic characteristics of bone resorption has shown that OXPHOS decreases as bone resorption increases, in contrast to the effects of MYC on osteoclast differentiation [70]. This may be explained by the fact that the energy metabolism of osteoclasts differs depending on the stage of differentiation and bone resorption activity.

**Table 1:** The altered functional subunits in degenerative joint diseases.

Subunit name	Gene origin	Cofactors	Influencing factors	Modulated cells	Possible functions	Ref
**Complex I**						
**NDUFB8**	Nuclear	NA	Aging, Osteoarthritis	Osteoblasts, Osteoclasts, Chondrocytes	Promote osteogenic differentiation, Slow the progression of osteoarthritis	[85, 88, 104]
**NDUFA13**	Nuclear	NA	Aging	Osteoblasts	Promote osteogenic differentiation	[88]
**NDUFS4**	Nuclear	NA	Aging	Osteoclasts	Promote the activation of pro-inflammatory macrophages, Inhibit osteoclasts	[99]
**NDUFC1**	Nuclear	NA	Notch signaling	Osteoblasts	Promote osteogenic differentiation	[94]
**Complex II**						
**SDHA**	Nuclear	FAD	Aging	Osteoblasts	NA	[88]
**SDHB**	Nuclear	2Fe-2S, 4Fe-4S, 3Fe-4S clusters	Aging	Chondrocytes	Slow the progression of osteoarthritis	[104]
**Complex III**						
**UQCRC1**	Nuclear	NA	Microgravity, Aging	Osteoblasts, Chondrocytes	Promote osteogenic differentiation, Slow the progression of osteoarthritis	[103, 109]
**UQCRC2**	Nuclear	NA	Osteoarthritis	Chondrocytes	NA	[104]
**Complex IV**						
**COX1**	Mitochondrial	Cu_B_, Mg^2+^, Haem a/a_3_	Aging	Osteoblasts	Maintenance of osteogenic differentiation	[127]
**COX2**	Mitochondrial	Cu_A_	Aging	Osteoblasts	Maintenance of osteogenic differentiation	[127]
**COX4I2**	Nuclear	NA	SIRT3 signaling	Chondrocytes	Maintenance of cartilage integrity	[133]
**Complex V**						
**ATP5A**	Nuclear	NA	Osteoarthritis	Chondrocytes	NA	[104]

Chondrocytes are found in articular cartilage and exhibit high levels of glycolysis. Despite the presence of oxygen, anaerobic glycolysis and lactic acid production are involved in the respiratory metabolism of articular cartilage [156]. Due to the lack of blood vessels and low oxygen levels, cartilage is considered a hypoxic tissue; as such, its mitochondrial metabolism has not been extensively studied. Recent studies have shown that the OXPHOS system produces 25% of the ATP in cartilage, while O_2_ is mainly sourced from synovium. Additionally, there is evidence suggesting that mitochondrial OXPHOS metabolism is at the core of extracellular matrix calcification [157]. In the development of OA, available data suggest that there is a decrease in the number of chondrocyte mitochondria, which leads to decreased ATP levels due to reduced OXPHOS within each chondrocyte. This decrease in ATP production is compensated for by an increase in glycolysis. When the chondrocyte MRC is blocked, intracellular ATP levels and mitochondrial ΔΨ decrease, resulting in decreases in proteoglycan levels and cartilage function. Furthermore, research has revealed that the TFAM expression level in OA chondrocytes is low. However, silencing of TFAM in chondrocytes further reduced the mtDNA content and mitochondrial quality, leading to inhibited expression of CI-V and ultimately resulting in cartilage decompensation [103]. Therefore, MRC-mediated OXPHOS also plays a critical role in chondrocyte metabolism.

## 5. The MRC and ROS generation in bone metabolism

Numerous studies have demonstrated that cell survival is heavily reliant on functional mitochondria. Dysregulation of intracellular ROS is a primary cause of mitochondrial damage [158, 159]. Interestingly, mitochondrial damage leads to changes in various cellular functions, such as OXPHOS and the intracellular redox system, and triggers a series of chemical reactions that generate harmful oxygen free radicals, ultimately disrupting cell growth and metabolism. The resulting cellular damage can contribute to the development of bone-related diseases and senescence [160].

ROS are a group of molecules that include singlet oxygen, superoxide anion, and hydrogen peroxide [161]. Recent research has demonstrated that ROS actively regulate biological processes at all levels of an organism [162]. In mitochondria, ROS are primarily produced through two pathways: electron leakage from the electron transport chain in normal cells and damage to the electron transport chain in damaged cells [163]. Under normal physiological conditions, a portion of the electron transport chain in the IMM becomes detached, resulting in the reduction of approximately 1-2% of oxygen to superoxide anion. This anion damages the mitochondria and leads to the production of additional ROS and reactive nitrogen species [164]. Superoxide anions react with manganese peroxide dismutase to produce hydrogen peroxide. This hydrogen peroxide is then further processed by enzymes, such as glutathione peroxidase and catalase, which reduce it to water. However, some of the hydrogen peroxide is converted into highly active hydroxyl radicals through iron chelation [165-167]. The peroxides produced by these pathways can cause mitochondrial dysfunction and cell damage either directly or indirectly.

Mitochondria are the primary source of overproduction of intracellular ROS, which can cause damage to the mitochondria. The pathways through which ROS cause mitochondrial damage can be broadly divided into two categories. First, ROS can affect the activity of MRC complexes, which can reduce the efficiency of MRC, thereby affecting mitochondrial OXPHOS and resulting in reduced intracellular ATP synthesis. Mild disruption in the cell will produce an increased amount of ROS that, if not promptly eliminated, accumulate and cause additional damage to the mitochondria, ultimately resulting in the loss of cell function [168, 169]. Second, excessive ROS production can alter the permeability of the mitochondrial membrane, resulting in the release of cyto-c into the matrix. This triggers the activation of caspases, leading to cytotoxicity and ultimately resulting in cell apoptosis or necrosis. Furthermore, excessive production of free radicals can disrupt the transcription and translation of mtDNA, leading to various abnormalities, such as point mutations, deletions, or insertions. This ultimately hinders the synthesis of mtDNA-encoded proteins, causing reduced mtDNA levels and mitochondrial numbers, and metabolic disorder within the cell. When a cell is unable to compensate for a large amount of ROS production, the ROS attack cellular biological macromolecules, including proteins, lipids, and nucleic acids. This can lead to a reduction in intracellular antioxidant enzyme levels and the generation of a significant amount of malondialdehyde, ultimately causing oxidative damage [170, 171]. In summary, ROS plays crucial roles in activating multiple signal transduction pathways within cells, resulting in a wide range of cellular biological responses.

CI is the primary transporter of electrons and the subsequent production of superoxide in mitochondrial membranes. A deficiency in CI can lead to an increase in ROS production and a decrease in antioxidant defense, ultimately impairing mitochondrial function [172]. One of the key auxiliary subunits of CI is NDUFS6, which downregulated during aging of BMSCs. Furthermore, NDUFS6 deficiency in BMSCs has been shown to impair CI function and activate the ROS/p53/p21 signaling pathway, ultimately leading to cellular aging. Mito-tempo is a superoxide dismutase (SOD) mimic that targets mitochondria and has the ability to effectively eliminate superoxide and alkyl radicals. In a recent study, treatment of NDUFS6^-/-^ BMSCs with Mito-Tempo blocked the generation of ROS and the expression of senescence-related markers [173]. Notably, mutations in NDUFS4 have been identified as a significant cause of early-onset Leigh syndrome. In fibroblasts with mitochondrial NDUFS4 deficiency, researchers observed increased levels of intracellular ROS and dysregulation of antioxidant proteins. Additionally, all cell lines deficient in CI exhibited reduced basal and maximal respiratory capacity. However, when these cell lines were cocultured with BMSCs, researchers observed significant improvements in bioenergetic profiles and oxidation levels [174]. Additionally, the construction of mice with global NDUFS4 deletion resulted in systemic inflammation and osteosclerosis. Loss of NDUFS4 in the hematopoietic system leads to a shift in the intrinsic lineage from osteoclasts to macrophages, further decreasing osteoclast lineage commitment by activating NDUFS4^-/-^ macrophages through overexpression of ROS [99]. Fe-S protein plays a crucial role in the respiratory chain. Deferoxamine (DFO), an iron chelating agent, can inhibit CI, CII and CIII. However, DFO-mediated blockage of the respiratory chain did not stimulate osteoclast differentiation through the RANKL-ROS-mitogen-activated protein kinase (MAPK) pathway. Instead, it interfered with osteoclast differentiation and bone resorption by inhibiting MAPK [175]. Inhibition of the respiratory chain by DFO in the presence of ROS also inhibited osteoclast function, suggesting that abnormal energy metabolism, or other factors caused by the mitochondrial complex, may have a greater impact on the regulation of bone phenotypes.

Compared with CI, CII produces significantly less ROS and some researchers have proposed that CII does not generate ROS at all. However, a follow-up study revealed that antimycin A can bind to coQ, causing electrons at CII to aggregate and induce ROS [105, 176]. Quinlan *et al*. showed that simultaneous inhibition of CI and CIII prevented electrons from flowing to CIII, instead reversing them to CII through coQ [177]. The potential of the succinate dehydrogenase site is thought to decrease, leading to the generation of ROS by CII. However, recent research has significantly improved our understanding, revealing flavin mononucleotides as the primary source of ROS in CII. In isolated mitochondria, the production of ROS by CII is highly regulated by the succinate concentration. Specifically, there is a bell-shaped response, i.e., ROS production is optimized at succinate concentrations that are neither too high nor too low. When the substrate NAD^+^ of mitochondrial CI is restricted, electrons can be transferred from CII to CI with the assistance of the CII substrate succinate. This process can also enhance the activity of CI, leading to increased oxygen production [168, 169]. While the mechanism of ROS production by CII is currently being clarified, its roles in bone metabolism and total mitochondrial ROS production under physiological conditions have yet to be determined. In OA chondrocytes, the decreased activity of MRC complexes results in decreased mitochondrial bioenergy reserves and negatively impacts the cellular redox balance, which is dependent on the physiological production of low levels of ROS [178]. Restoration of mitochondrial function through the reduction of ROS levels within mitochondria has emerged as a promising therapeutic target for OA.

The respiratory chain has two primary sites that release superoxide: CI and CIII. Under conditions of hypoxia, the primary source of ROS is damage to mitochondrial CIII. Within the CIII system, the Qo oxidation site plays a critical role in the production of intracellular ROS [179, 180]. Antimycin A can impede the transfer of CIII electrons from cytochrome B566 to quinone reduction site "Qi", causing the accumulation of semiquinone in the oxidation site Qo, which in turn facilitates the conversion of O_2_ to superoxide [181]. MT3, a member of the metallothionein protein family, takes part in promoting osteoblast differentiation by reducing oxidative stress levels and promoting the expression of Runx2/Osterix/Dlx5. Antimycin A is known to inhibit CIII of the electron transport system and has been utilized as an ROS generator in biological systems. When combined with MT3-overexpressing PlasMIDs, there was a significant decrease in osteoblast viability, which was accompanied by a surge in intracellular ROS levels [182]. Furthermore, antimycin A has been shown to trigger a disruption in the structure of mitochondrial CIII in osteoblasts [119], resulting in overexpression of ROS and ultimately leading to osteogenesis disorder. However, numerous bioactive substances have been identified that effectively reverse this phenomenon and restore stability to redox homeostasis in osteoblasts [116, 118].

CoQ is a quinone compound that is ubiquitous in cells throughout the body. It serves as a vital component of the MRC and a cofactor in the mitochondrial electron transport chain, where it moves within the IMM and facilitates the transfer of electrons from CI and CII to CIII [183, 184]. CoQ10 is involved in cellular energy metabolism and circulates between oxidative and reductive forms. Its unique function includes the transfer of protons outside the mitochondrial membrane and the transfer of electrons from the primary substrate to the oxidase system, which results in the formation of a gradient. This gradient is utilized by protons returning to the mitochondria via the enzymes that make ATP, driving ATP formation. Reportedly, CoQ10 activates the PTEN/PI3K/AKT pathway in a dose-dependent manner, leading to significant increases in the proliferation and osteogenic differentiation of BMSCs, and the expression of osteogenic markers [185]. Moon *et al*. discovered that utilization of CoQ10 as an antioxidant at a biological dose significantly reduced the formation of TRAP-positive polykaryotic osteoclasts. This was achieved by downregulating MAPK and IKBα signals [186]. Further mechanistic studies suggested that the antagonistic effect of CoQ10 on osteoclasts may be exerted through the downregulation of intracellular inflammatory mediators and lipid oxidation factors. Additionally, CoQ10 supplementation has been found to increase the levels of antioxidant proteins, including SOD and catalase, as well as molecular compounds that promote apoptosis [187]. Furthermore, active CoQ10 supplementation can regulate osteogenesis and osteoclast coupling, and may have positive impacts on postmenopausal and spinal cord injury-induced osteoporosis, periodontal disease, and rheumatoid arthritis [186, 188]. In a study of chondrocytes treated with CoQ10, it was observed that CoQ10 effectively prevented cartilage inflammation and matrix degradation by inhibiting MAPK signaling [189]. CoQ plays prominent roles in regulating the connection between osteoblasts and osteoclasts, and in maintaining the integrity of cartilage. Supplementation with CoQ may be a promising option for bone joints undergoing age-related and functional degeneration due to epigenetic alterations or loss of protein stability.

**Figure 4.**
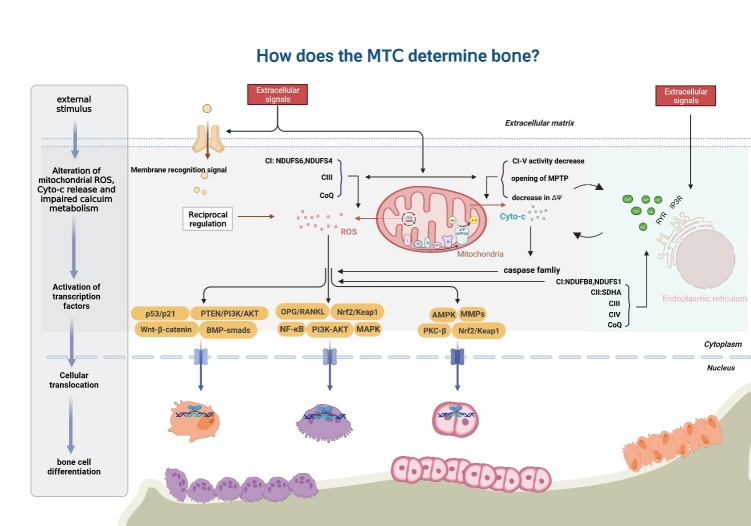
**The MRC and ROS generation in bone metabolism**. Cells stimulated by external factors exhibit disrupted redox homeostasis, resulting in the inhibition of IMM transmission and the production of a large amount of ROS. The viability of the cellular mitochondrial complex is reduced, leading to opening of the cellular mitochondrial permeability transition pore (MPTP), which releases cyto-c and triggers the caspase-induced mitochondrial apoptotic pathway. Additionally, abnormal conditions such as hypoxia, and aging can cause ER stress and destabilize calcium influx. The excessive production of ROS and the interaction of apoptosis and impaired calcium homeostasis trigger a cascade of downstream signals in the cytoplasm, which affects the transcription and translation of related molecules, ultimately leading to changes in the differentiation of bone-related cells.

## 6. The MRC and apoptosis in bone metabolism

For healthy cells to remain vital, neighboring aged or overly injured cells must undergo self-destruction. This process, known as apoptosis, is essential for physiological bone transformation, repair, and regeneration [190]. The mitochondria act as a guiding hand in regulating cellular activities, serving as both the center of cellular OXPHOS and the control center for cell apoptosis. Recent studies have identified several substances within mitochondria that play crucial roles in apoptosis, including cyto-c, apoptosis-inducing factor, Ca^2+^, and ROS. Of these, cyto-c release is a pivotal step in the apoptosis process [191, 192]. Various signals ultimately converge at the mitochondria, where they can either activate or inhibit certain events. Furthermore, the loss of mitochondrial function may be linked to the creation of short-lived free radical intermediates during oxygen transfer. These intermediates may transmit signals that trigger apoptosis. Collectively, mitochondria regulate apoptosis through specific signal transduction pathways. Understanding apoptosis-specific triggering mechanisms is a crucially important topic of bone metabolism research.

The size of the osteoblast population is determined by the balance between osteoblast proliferation and apoptosis. Osteoblasts with typical apoptotic features are not commonly found *in vivo*, but can be found in fracture calluses and at the anterior end of sutures of the developing mouse skull [193, 194]. It is estimated that 60-80% of the osteoblasts initially clustered in the absorption pit undergo apoptosis. Apoptosis in osteoclasts is typically characterized by cell contraction, chromatin aggregation, nuclear fragmentation, and intense TRAP staining. The importance of osteoclast apoptosis in bone disease has been established through various genetically modified animal models. Decreased osteoclast apoptosis can often result in increased bone loss, which occurs under the estrogen-deficient conditions following ovariectomy [195]. This phenomenon is also observed in OA [196]. The involvement of the Fas signaling pathway has been confirmed in osteoclast apoptosis in both mice and humans. Activation of the receptor mediates the mitochondrial release of cyto-c and the activation of caspases [197]. The deterioration of human articular cartilage with aging may be attributable to a decrease in the number of chondrocytes, which are unable to properly regenerate and reshape the cartilage. As OA progresses, cartilage shows signs of chondrocyte apoptosis, including a depletion of cells and lacunar emptying [76]. The malfunctioning of MRC activity in chondrocytes, and the resulting disturbance in the movement of electrons, can lead to the generation of ROS, which may cause an increase in the natural apoptosis of chondrocytes in OA [198].

Cyto-c is a water-soluble pigment found on the cytoplasmic side of mitochondria, where it freely diffuses and plays a crucial role in electron transport between CIII and CIV. Its principal activity involves the oxidation of tissues in the presence of enzymes, which restores the original rapid enzyme-promoting effect [199]. During anoxia, when the permeability of the cell membrane increases, cyto-c is likely to enter cells and mitochondria, enhancing cell oxidation and improving oxygen utilization. Cyto-c is not only an electron transporter in the respiratory chain, but also an important protein regulating the apoptotic pathway. It is generally believed that mitochondrial injury leads to cyto-c release from the mitochondrial membrane space into the cytoplasm, inducing apoptosis through a caspase-dependent pathway. Studies have shown that IFN-γ and TNF-α induce osteoblast apoptosis through cyto-c release, caspase activation, and BCL-2 downregulation in damaged mitochondria. Additionally, AlCl_3_ was found to induce the appearance of osteoblast morphology and an increase in the apoptosis rate, which was accompanied by mitochondrial ΔΨ depolarization, cyto-c release from the mitochondria to the cytoplasm, and caspase 9 activation [200]. It is evident that mitochondrial release of cyto-c negatively impacts osteoblast differentiation. The use of traditional Chinese medicine has led to the discovery of numerous herbal monomers that are involved in mitochondrial apoptosis. These monomers mediate the release of cyto-c and regulate the differentiation and functional activity of osteoblasts [201-203]. Regarding the regulation of osteoclasts, it has been established that natural cyto-c and hydrogen peroxide derived from mitochondria induce osteoclast apoptosis. In a study by Angireddy *et al*., mitochondrial stress was induced in macrophages via KD of a subunit of CIV. They demonstrated that IVi1 KD cells exhibited elevated levels of mitochondrial ROS, heightened cellular glycolysis and increased potential for osteoclast differentiation [204]. In mouse aging and OA models, significant reductions in lysosome acidification induced apoptosis in chondrocytes, which was mediated by mitochondrial damage caused by BAX and cyto-c release [205]. Exposure of chondrocytes to oxidative stress induced by H_2_O_2_ resulted in increased release of cyto-c and inhibition of SOD-2 activity. This led to mitochondrial damage, which was found to disrupt energy metabolism and ultimately trigger chondrocyte apoptosis through the mitochondrial pathway [206].

## 7. The MRC and calcium transport in bone metabolism

Although the concentration of Ca^2+^ is less than 0.1% of the total amount, it plays a vital physiological role in maintaining homeostasis. Ca^2+^ serves as the most prevalent signal transducer, reversibly binding to numerous intracellular proteins, and also functions as a typical second messenger. Ca^2+^ binding to calmodulin activates kinases that dephosphorylate specific proteins, leading to diverse biological effects in various cell types. Additionally, Ca^2+^ is a critically important accessory factor for enzymes. The activities of many enzymes rely on Ca^2+^ binding, which remains unaffected by changes in extracellular Ca^2+^ concentration [207, 208]. Endogenous Ca^2+^ signaling is active in bone metabolism and remodeling. The involvement of various Ca^2+^ channels and calmodulin kinases in the life cycle of bone cells and biological metabolism through molecular cascade signals is well established. Intracellular Ca^2+^ acts as a key factor in the differentiation of osteoblasts, osteoclasts, and chondrocytes, and its redistribution regulates cell differentiation, proliferation, migration, and other processes [209-211]. Several types of Ca^2+^ channels exist on the cell membrane structure, including: ryanodine receptors, inositol-1, -4, and -5-trisphosphate receptors, store-operated Ca^2+^ channels, stretch-activated Ca^2+^ channels, voltage-gated Ca^2+^ channels (VGCCs), and members of the transient receptor potential superfamily [212]. Inside the cell, mitochondria take up Ca^2+^ mainly through the mitochondrial Ca^2+^ uniporter. The Ca^2+^ release-activated and load-activated Ca^2+^ channels on the ER work together to maintain Ca^2+^ homeostasis [213]. Alterations in these Ca^2+^ channels have also been shown to exacerbate pathological processes in MRC disorders and lead to a cascade reaction.

NADH and flavin adenine dinucleotide (FADH_2_) transfer H^+^ and electrons through successive enzymatic reactions to release energy. The MRC transfers electrons and H^+^, resulting in the formation of an electrochemical proton gradient on either side of the IMM, creating a ΔΨ difference. This ΔΨ provides a great driving force for Ca^2+^ uptake by mitochondria [214]. Therefore, normal operation of the MRC is closely linked to Ca^2+^ transport, and blockage of the MRC leads to an imbalance in Ca^2+^ homeostasis. Ca^2+^-related apoptosis, oxidative stress, and OXPHOS are also affected [215]. There are a variety of Ca^2+^ transport structures on the IMM. Although the link between mitochondria and Ca^2+^ has been well established in the physiology and pathology of cells, the regulation of Ca^2+^ transport by the MRC has not been thoroughly investigated. What is known is that the MRC is the main site of ROS generation, Ca^2+^ signaling can influence ROS production, and increased ROS production can also alter Ca^2+^ distribution [216]. In this paper, our focus is on the impacts of MRC blockage on Ca^2+^ transport and signaling in osteogenesis, osteoclasts, and chondrocytes, with the aim of providing insight into the chain reaction triggered by Ca^2+^.

Defects in the chain reaction of CI, which is the largest complex in the OXPHOS system, is a common subject of discussion. When exposed to hypoxia, CI undergoes conformational changes that result in the release of Ca^2+^ from the mitochondria due to the dissolution of calcium phosphate precipitates. These precipitates are necessary for CI to exchange Na^+^ and Ca^2+^. The inactivation of CI under hypoxia has revealed that the Cys39 residue of the ND3 subunit acts to maintain the Na^+^/Ca^2+^ exchanger [217]. The CI inhibitor rotenone has been found to decrease Ca^2+^ oscillations, likely by hindering the MRC and impairing the mitochondrial amplification of Ca^2+^ signals [218]. Consequently, depolarization of the ΔΨ results in a decline in ATP production and insufficient ATP supply to the Ca^2+^-ATPase, ultimately leading to an overload of intracellular Ca^2+^. Metformin, a classical hypoglycemic agent, is also intriguing owing to its potential to stimulate osteoblast proliferation and differentiation [219]. Studies have demonstrated that metformin inhibits CI and activates AMPK by elevating the AMP/ATP ratio, resulting in osteoprotective effects through the phosphorylation of downstream targets of AMPK [220]. Despite these findings, the effects of AMPK and Ca^2+^ fluctuations on osteoblasts remain unexplored. CCN6, also known as Wnt-induced signaling protein 3, has been linked to the skeletal disease known as progressive pseudorheumatoid dysplasia [221]. This disease is characterized by progressive cartilage loss and irregular bone growth. The insulin growth factor (IGF) binding protein domain of CCN6 is believed to play a role in this disease by modulating IGF1, and increased ROS production is considered to be the hallmark process of cartilage hypertrophy. Additionally, moderate depletion of CCN6 results in increased assembly and activity of CI, as evidenced by increases in the NDUFB8 and NDUFS1 subunits [222, 223]. These findings suggest that CCN6 is an essential component of CI, and that mutations in CCN6 can severely impair mitochondrial function and cell survival. CCN6 induces increased production of ROS and depletion of ATP, resulting in Ca^2+^ overload. However, this process can be inhibited by Nrf2. Subsequent experiments have shown that such Ca^2+^ signaling is associated with cell matrix mineralization in chondrocytes [224].

CII is an essential enzyme in oxidative metabolism and the citric acid cycle. Cells that lack CII often experience a decline in mitochondrial function, disruptions in ΔΨ, and Ca^2+^ overload, which can ultimately result in cell death [225]. Under respiratory chain blockade, the expected changes in Ca^2+^ uptake triggered by alterations in ΔΨ are reversed [225]. Mitochondrial Ca^2+^ and ER Ca^2+^ are closely linked, and an increase in ER Ca^2+^ leakage occurs in CII deficiency, ultimately resulting in mitochondrial Ca^2+^ overload [226]. Additionally, Hipps *et al*. discovered a statistically significant difference in the SDHA subunit of CII in the respiratory chain state of osteoblasts from osteoporotic patients [88].

CIII is made up of 11 polypeptides and contains two revitalization centers: oxidation center Q_o_ and reduction center Q_i_. Antimycin A and myxothiazol have been discovered to inhibit these two centers and regulate VGCCs, respectively [227]. Although their binding centers differ, studies have revealed that inhibiting CIII function with both antimycin A and myxothiazol results in increased ROS production. In one study, treatment with antimycin A reduced the peak amplitude of the voltage-gated Ca^2+^ current in osteoblastic MC3T3-E1 cells and increased bone resorption cytokines. This finding is consistent with the previous section, which discussed how antimycin A can lead to an increase in mitochondrial Ca^2+^ release through ROS [228]. Increases in ROS generation and intracellular Ca^2+^ concentration were observed in osteoblasts following CIII blockade; however, no abnormal increase in ROS was found in osteoblasts following a control intervention that induced Ca^2+^. Ca^2+^ promotes bone resorption by releasing the bone resorptive cytokines IL-6 and TNF-α from osteoblasts, and by activating osteoclasts that destroy bone tissue. Antimycin A has been found to trigger increased ROS production, which leads to decreased OPG expression and increased RANKL expression [228].

In the context of CIV, alterations in mitochondrial Ca^2+^ levels have been observed in response to the inhibition of the respiratory chain by NO. Previous studies have shown that the introduction of exogenous NO can trigger the release of Ca^2+^ from mitochondria, while the disruption of CIV may impact the regulation of Ca^2+^ flux between mitochondria and the ER [229, 230]. Research has suggested that CIV blockade may lead to an increase in intracellular Ca^2+^ concentration due to an increase in VGCC-mediated Ca^2+^ influx. Additionally, it has been found that an increase in ROS release from CIII and mitochondrial membrane hyperpolarization can lead to an increase in cell membrane Ca^2+^. Apart from these findings, there is little research on how CIV specifically regulates Ca^2+^.

Ivan Bogeski and colleagues discovered that hydroxy CoQ exhibits a remarkable ability to bind Ca^2+^, suggesting that it could be a key player in transmembrane transport processes. The putative mechanism involves CoQ undergoing structural modifications and generating hydroxyl derivatives in response to exposure to alkaline environments or interaction with CYP450 enzymes. Specifically, the CoQ quinone ring binds Ca^2+^ in its reduced form and then releases it in its oxidized form. Additionally, hydroxy CoQ may have a role in scavenging ROS, buffering Ca^2+^, and facilitating Ca^2+^ transport across the IMM [231]. Calcineurin is also a target protein activated by Ca^2+^/calmodulin. In the context of CIV blockade, there is a noticeable and sustained rise in cytosolic Ca^2+^ concentration resulting from the loss of ΔΨ. This increase activates calcineurin, along with downstream transcription factors, such as NFκB, NFAT, CREB, and CCAAT/enhancer-binding protein δ (C/EBPδ) [232]. Additionally, silencing of the IVi1 and Vb subunits of CIV produces a form of retrograde signaling from the mitochondria to the nucleus (MtRS), which ultimately promotes osteoclast differentiation by influencing M1 polarization of macrophages [204]. No significant increases in the nuclear localization of MtRS, C/EBPδ, p50, or c-Rel were observed under Vb KD. By contrast, KD of IVi1 led to high-level expression of IL-2b, IL-6, IL-10, and TNF-α, and a significant enhancement in the ability of macrophages to differentiate into osteoclasts [233]. However, it is worth noting that ROS production was also increased in the presence of CIV injury. There have been numerous reports on ROS-RANKL pathway-mediated osteoclast formation, which is enhanced by CIV impairment, and upregulation of osteoclastogenesis. Alterations in mitochondrial ΔΨ and electron transport coupled to ATP synthesis upregulate lysine receptor-gated Ca^2+^ channels and increase cytosolic Ca^2+^. Activation of calcineurin, a Ca^2+^-calmodulin-responsive phosphatase, affects massive production of proinflammatory cytokines and enhances osteoclast-related gene expression [204]. However, since Ca^2+^ transients and ROS generation are likely to coexist in their co-regulation of osteoclasts, the possibility of cross-reaction needs further study.

## 8. The MRC and mitophagy in bone metabolism

Mitophagy is a crucial process for maintaining the integrity of mitochondria, serving as a form of mitochondrial quality control. It plays a vital role in regulating the number and function of mitochondria by eliminating damaged organelles and excess proteins, thus reducing cellular stress caused by harmful stimuli [234]. Under pathological conditions, mitophagy promotes cellular homeostasis by minimizing intracellular oxidative damage to impaired mitochondria. Cells experiencing oxidative stress activate the mitophagy pathway to remove dysfunctional mitochondria with damage that has surpassed the MMP of the IMM [235, 236]. This helps to preserve the integrity and repair capacity of mtDNA in response to stress. Emerging evidence suggests that abnormal levels of mitophagy disrupt bone metabolic homeostasis and act in bone metabolic disorders.

The feasibility of bone-associated cells regulating mitophagy via the MRC is supported by correlative evidence. Electron leakage from the MRC increases intracellular levels of ROS, and mitophagy plays a role in maintaining tissue homeostasis by reducing the intracellular ROS produced by damaged mitochondria. It also recycles energy by limiting the energy demand of ineffective organelles and generating ATP from degradation, both under physiological and pathological conditions. Damaged mitochondria release ROS and apoptotic factors, leading to apoptosis in bone-associated cells [237, 238]. However, mitophagy can degrade damaged mitochondria, thereby protecting cells from apoptosis. Additionally, mitochondria affect intracellular Ca^2+^ signaling by providing ATP for Ca^2+^ transporter proteins. The levels of mitophagy also influence intracellular Ca^2+^ signaling [239]. Disturbances in mitophagy can have detrimental effects on mitochondrial homeostasis, cellular energy metabolism, and the physiological functions of bone-associated cells.

Mitophagy plays a crucial role in maintaining the survival of osteoblasts during osteogenic differentiation [240]. The PTEN-induced kinase 1 (PINK1)/Parkin pathway is the most extensively studied mechanism for inducing mitophagy. Under MMP impairment, PINK1 is unable to enter the IMM, leading to its accumulation on the cytoplasmic surface of the OMM. PINK1 accumulation triggers the recruitment and activation of Parkin, which then changes its spatial conformation into that of an activated E3 ubiquitin ligase, subsequently ubiquitinating proteins on the mitochondria. PINK1 and Parkin interact to regulate mitophagy and maintain mitochondrial mass [38]. Studies have revealed that advanced oxidation protein products (AOPPs) can induce the production of ROS and lead to depolarization of the ΔΨ. This, in turn, triggers the mitochondria-dependent intrinsic apoptotic pathway in osteoblasts. Clearing excess ROS and damaged mitochondria is essential for reversing AOPP-induced apoptosis. In this experiment, rapamycin was observed to further activate PINK1/Parkin-mediated mitophagy in AOPP-stimulated MC3T3-E1 cells. This activation significantly reduced AOPP-induced apoptosis by eliminating ROS and damaged mitochondria [241]. Other ubiquitin-dependent pathways do not rely on Parkin. PINK1 recruits autophagy receptor proteins (such as NIX, BNIP3 and FUNDC1) directly to mitochondria through ubiquitin phosphorylation. These receptor proteins then recruit LC3, which enables the autophagosome to engulf the mitochondria [242]. In a study investigating the underlying mechanism of glucocorticoid-induced necrosis of the femoral head, mouse long bone osteoblast Y4 cells were exposed to glucocorticoid under hypoxic conditions. The study found that this exposure restored cellular mitophagy and an increase in apoptosis. However, when hypoxia-inducible factor (HIF)-1α was overexpressed in a hypoxic environment, it was able to resist glucocorticoid-induced apoptosis through its downstream marker BNIP3. This was primarily achieved by reducing the inhibition of hypoxia-induced mitophagy caused by glucocorticoids, thus protecting osteoblasts from apoptosis [243]. Chen et al. discovered that Apelin-13, an adipokine produced by adipocytes, improved oxidative stress by activating mitophagy in BMSCs. This activation led to the restoration of osteogenic function through AMPK-α phosphorylation [244]. In a similar study, Liu et al. demonstrated that mitophagy provided a protective effect against aluminum-induced osteoblast dysfunction. Therefore, it can be concluded that the maintenance of mitophagy is essential for the functional regulation of osteoblasts [245].

The effects of mitophagy on osteoclast differentiation have not been extensively studied. However, intracellular mitophagy levels are known to increase during osteoclast differentiation [246]. While recent reports suggest that mitophagy and the Parkin pathway play roles in the entire process of osteoclastogenesis, the impact of regulating mitophagy levels on osteoclast metabolism remains unclear. Sarkar *et al*. discovered that epigallocatechin-3-gallate, a natural plant product, suppressed the mRNA and protein expression of genes associated with mitophagy. This inhibition was observed *in vitro*, in primary osteoclast differentiation of bone marrow cells. Epigallocatechin-3-gallate also reduced the levels of mitochondrial ROS and ATP formation during osteoclast differentiation [247]. However, a study by Yao *et al.* found that Guizhi Shaoyao decoction, an herbal formula, promoted mitophagy in osteoclasts through PINK1/Parkin signaling, resulting in the inhibition of osteoclast differentiation. When PINK1 expression in osteoclasts was reduced, the inhibitory effect of Guizhi Shaoyao on osteoclastogenesis was eliminated [248]. Regulation of mitophagy levels can impact macrophage inflammatory phenotypes. Research has revealed that the activation of macrophage NLRP3 inflammatory vesicles, which leads to the expansion of osteoclasts by inflammatory macrophages, was significantly blocked by pharmacological inhibition of STAT3. This blocking process primarily occurs through the induction of PINK1-dependent mitophagy, which then reverses the collapse in mitochondrial ΔΨ and inhibits the release of mitochondrial ROS [249].

As individuals age, imbalances in the catabolism and anabolism of the extracellular matrix of cartilage lead to the development of fragile joints that are more susceptible to external damage. Research studies have revealed that, while autophagy-related proteins are highly expressed in clusters of human chondrocytes, their expression decreases in older populations. Senescence also significantly impacts the survival of chondrocytes through autophagy, thereby contributing to the development of OA [250]. Furthermore, mitochondrial dysfunction has been identified as a potential cause of cartilage degeneration, with recent studies highlighting the importance of mitophagy in chondrocyte metabolism [251]. Senescent chondrocytes exhibit increased lipid accumulation and fatty acid oxidation. However, reducing the activity of the fatty acid oxidation rate-limiting enzyme Cpt1a has been shown to inhibit cellular ROS levels and promote the mitochondrial autophagy pathway, which helps maintain mitochondrial homeostasis [252]. Additionally, pharmacological activation of mitophagy in chondrocytes has demonstrated significant protective effects against mitochondrial dysfunction, thereby preventing oxidative stress. One report indicated that mitochonic acid-5 enhances the activity of SIRT3 and promotes mitophagy through the Parkin-dependent pathway, eliminating depolarized mitochondria and providing protection to chondrocytes [253]. Additionally, curcumin has been found to activate mitophagy by activating the AMPK/PINK1/Parkin pathway, exhibiting chondroprotective effects in OA [254].

## 9. Conclusion and perspectives

Mitochondria have become a prominent subject of biomedical research because of their crucial involvement in the process of aging and the emergence of various human diseases. The MRC, which is composed of five enzymatic complexes and two mobile electron carriers, is responsible for aerobic respiration and the production of most cellular ATP through OXPHOS. Mitochondria are also a significant source of cellular ROS and actively regulate intracellular calcium homeostasis and apoptosis. When the MRC is inhibited, the proton gradient in the IMM collapses, leading to a loss of mitochondrial ΔΨ and the production of ROS. Disruptions in the breakdown of ROS or ΔΨ can result in the opening of the mitochondrial permeability conversion pore. This, in turn, can impair energy metabolism and cause oxidative stress and damage. In severe cases, cell death may occur as a result of this dysfunction. To counteract the excessive accumulation of free radicals, cells initiate mitophagy, a process that removes depolarized mitochondria and reduces intracellular levels of ROS. This mechanism plays a crucial role in maintaining tissue homeostasis.

Intracellular energy metabolism plays a crucial role in regulating the cascade amplification mechanism of various molecular signals. This regulation ultimately controls the processes of gene transcription and translation, which in turn regulates the cell phenotype. While there is limited research on the regulation of the MRC complex in bone cells, it is evident that the energy regulation facilitated by the MRC has a significant impact on the overall physiology of bone. Several studies have demonstrated that inhibiting the MRC pharmacologically can block the continuation of the electron transport chain and osteoclast differentiation. This inhibition primarily works by reducing energy production and supply, although it may also be influenced by ROS overflow and Ca^2+^ flow. Chondrocytes rely on glycolysis for their function. Hence, disruptions in MRC transmission primarily impact cell growth, survival, and function by altering endogenous ROS and Ca^2+^ levels, as well as apoptotic signals. Although the MRC facilitates various biological processes, the predominant factor affecting its activity in bone is the primary form of energy metabolism utilized by the specific cell type. Currently, the regulation of MRC activity has shown potential in the treatment of metabolic diseases and is also gaining attention in orthopedic research. However, further studies are needed to refine the roles of the functional MRC subunits in bone metabolism.

The promotion of mitochondrial function in bone-associated cells is crucial for maintaining bone health and treating bone metabolism-related diseases. Natural aging-induced degeneration often leads to mitochondrial damage in bone-associated cells, resulting in decreased bone density and increased risk of fractures. In basic research, several strategies targeting mitochondrial function have been developed to prevent and delay bone problems. For instance, Hollenberg *et al*. reported that electromagnetic field therapy can activate mitochondrial OXPHOS and promote fracture repair [255]. Another approach involves mitochondrial transfer, which has shown potential in enhancing the function of BMSCs and promoting *in situ* bone defect repair [256]. Additionally, recent studies have indicated that osteogenic induction can stimulate mitochondrial fragmentation and secretion through CD38/cyclic ADP ribose signaling. Manipulating mitochondrial dynamics via KD of Opa1 or over-expression of Fis1 can increase mitochondrial fragmentation, leading to enhanced mitochondrial secretion and accelerated osteogenesis [257]. Given these clues, it is evident that enhancing mitochondrial function in clinical practice is a powerful approach for regulating bone health. By promoting mitochondrial function in osteoblasts, it is possible to improve bone structure and aid in fracture rehabilitation. While various clinical approaches have been explored, specific methods for promoting mitochondrial function in bone-associated cells are still being investigated, and include gene therapy, pharmacologic interventions, and physical exercise. The safety and effectiveness of these approaches will require extensive study and clinical trialing.

Research on the long-term effects and potential side effects of enhanced mitochondrial function on bone health is limited. However, preliminary information from existing studies suggests that optimization of mitochondrial function may accelerate fracture healing and bone tissue repair [258, 259]. It is important to note that excessive enhancement of mitochondrial function in bone metabolism may increase the risk of abnormal bone growth. Additionally, mitochondrial function is closely intertwined with the immune system, thus over-regulation of mitochondrial function may impact the functioning of immune cells [260]. Furthermore, drugs that are used to enhance mitochondrial function may interact with other medications, influencing their metabolism and effects [261]. Large-scale, long-term clinical studies of mitochondria-enhancing drugs in bone health are essential to better understand their mechanisms of action and to thoroughly assess the potential risks and benefits.

Bone remodeling is a multifaceted process that involves various bone cell types. Any disturbance in this process can cause an imbalance and result in diseases. To prevent bone resorption, it is reasonable to consider strategies that inhibit osteoclast activation by blocking the MRC. Although bone remodeling is complex, it is important to note that it is not solely dictated by a specific bone microenvironment or one intercellular interaction. Therefore, utilizing methods that only target these factors may have negative consequences, such as hindering bone formation and cartilage proliferation and differentiation, ultimately worsening the bone microenvironment. Regardless of the approach used in the respiratory chain, it is important to focus on targeted bone microenvironment therapy to reduce skeletal and extraskeletal side effects. In bone degeneration with progressive of senescence, MRC activity in bone-related cells decreases, leading to a deterioration of bone homeostasis through various pathways. To counteract this, upregulating mitochondrial complex activity to promote MRC electron transfer may be a feasible strategy to alleviate joint degeneration. Our research has shown that CoQ10 has great potential as a novel drug candidate with multiple regulatory factors. It can inhibit osteoclasts and promote osteogenesis while maintaining chondroproliferative activity, making it a promising option for further development. The development of clinical drugs that enhance CoQ10 activity also holds promise. Recent studies have shown that targeted delivery of mitochondria can have a positive impact on degenerative bone diseases. Because intrinsic mitochondrial homeostasis cannot be remodeled, the option to inject cells with new mitochondria may provide additional paths to healing.

Bone remodeling strategies hold promise for treating age-related degenerative bone diseases. It is crucial to develop novel drugs that adjust the bone remodeling process by targeting key regulatory molecules to modulate mitochondrial function. Additionally, the identification of growth factors and cytokines that influence mitochondrial function during growth, and thereby promote bone formation and repair, is of the utmost importance. Combining therapeutic approaches, such as pharmacotherapy, exercise, and nutritional support, may be necessary for age-related degenerative diseases. Furthermore, their impacts on regulation of bone homeostasis and mitochondrial function in bone-associated cells should be further explored. High-impact bone-remodeling drugs that can modulate mitochondrial function in bone-associated cells require clinical trials and long-term studies to confirm their effectiveness.

## References

[1] Hartmann H, Wirth K, Klusemann M (2013). Analysis of the load on the knee joint and vertebral column with changes in squatting depth and weight load. Sports Med, 43:993-1008. 23821469 10.1007/s40279-013-0073-6

[2] O'Leary SA, Paschos NK, Link JM, Klineberg EO, Hu JC, Athanasiou KA (2018). Facet Joints of the Spine: Structure-Function Relationships, Problems and Treatments, and the Potential for Regeneration. Annu Rev Biomed Eng, 20:145-170. 29494214 10.1146/annurev-bioeng-062117-120924

[3] Izzo R, Guarnieri G, Guglielmi G, Muto M (2013). Biomechanics of the spine. Part I: spinal stability. Eur J Radiol, 82:118-126. 23088879 10.1016/j.ejrad.2012.07.024

[4] Ma Y, Qi M, An Y, Zhang L, Yang R, Doro DH, et al. (2018). Autophagy controls mesenchymal stem cell properties and senescence during bone aging. Aging Cell, 17. 10.1111/acel.12709 PMC5770781 29210174

[5] Risbud MV, Shapiro IM (2014). Role of cytokines in intervertebral disc degeneration: pain and disc content. Nat Rev Rheumatol, 10:44-56. 24166242 10.1038/nrrheum.2013.160 PMC4151534

[6] Motta F, Barone E, Sica A, Selmi C (2023). Inflammaging and Osteoarthritis. Clin Rev Allergy Immunol, 64:222-238. 35716253 10.1007/s12016-022-08941-1

[7] Kim BJ, Koh JM (2019). Coupling factors involved in preserving bone balance. Cell Mol Life Sci, 76:1243-1253. 30515522 10.1007/s00018-018-2981-y PMC11105749

[8] Zhao CQ, Wang LM, Jiang LS, Dai LY (2007). The cell biology of intervertebral disc aging and degeneration. Ageing Res Rev, 6:247-261. 17870673 10.1016/j.arr.2007.08.001

[9] Zheng L, Zhang Z, Sheng P, Mobasheri A (2021). The role of metabolism in chondrocyte dysfunction and the progression of osteoarthritis. Ageing Res Rev, 66:101249. 33383189 10.1016/j.arr.2020.101249

[10] Ambrosi TH, Marecic O, McArdle A, Sinha R, Gulati GS, Tong X, et al. (2021). Aged skeletal stem cells generate an inflammatory degenerative niche. Nature, 597:256-262. 34381212 10.1038/s41586-021-03795-7 PMC8721524

[11] Chandra A, Lagnado AB, Farr JN, Schleusner M, Monroe DG, Saul D, et al. (2022). Bone Marrow Adiposity in Models of Radiation- and Aging-Related Bone Loss Is Dependent on Cellular Senescence. J Bone Miner Res, 37:997-1011. 35247283 10.1002/jbmr.4537 PMC9526878

[12] Dominguez LJ, Di Bella G, Belvedere M, Barbagallo M (2011). Physiology of the aging bone and mechanisms of action of bisphosphonates. Biogerontology, 12:397-408. 21695491 10.1007/s10522-011-9344-5

[13] Greenblatt MB, Tsai JN, Wein MN (2017). Bone Turnover Markers in the Diagnosis and Monitoring of Metabolic Bone Disease. Clin Chem, 63:464-474. 27940448 10.1373/clinchem.2016.259085 PMC5549920

[14] Montaner Ramón A (2020). Risk factors of bone mineral metabolic disorders. Semin Fetal Neonatal Med, 25:101068. 31862224 10.1016/j.siny.2019.101068

[15] Wawrzyniak A, Balawender K (2022). Structural and Metabolic Changes in Bone. Animals (Basel), 12. 35953935 10.3390/ani12151946 PMC9367262

[16] Suttapreyasri S, Koontongkaew S, Phongdara A, Leggat U (2006). Expression of bone morphogenetic proteins in normal human intramembranous and endochondral bones. Int J Oral Maxillofac Surg, 35:444-452. 16513322 10.1016/j.ijom.2006.01.021

[17] Tuckermann J, Adams RH (2021). The endothelium-bone axis in development, homeostasis and bone and joint disease. Nat Rev Rheumatol, 17:608-620. 34480164 10.1038/s41584-021-00682-3 PMC7612477

[18] Song S, Guo Y, Yang Y, Fu D (2022). Advances in pathogenesis and therapeutic strategies for osteoporosis. Pharmacol Ther, 237:108168. 35283172 10.1016/j.pharmthera.2022.108168

[19] Bucher CH, Schlundt C, Wulsten D, Sass FA, Wendler S, Ellinghaus A, et al. (2019). Experience in the Adaptive Immunity Impacts Bone Homeostasis, Remodeling, and Healing. Front Immunol, 10:797. 31031773 10.3389/fimmu.2019.00797 PMC6474158

[20] Li Q, Zhao Y, Deng D, Yang J, Chen Y, Liu J, et al. (2022). Aggravating Effects of Psychological Stress on Ligature-Induced Periodontitis via the Involvement of Local Oxidative Damage and NF-κB Activation. Mediators Inflamm, 2022:6447056. 35221795 10.1155/2022/6447056 PMC8866020

[21] Li Q, Yue T, Du X, Tang Z, Cui J, Wang W, et al. (2023). HSC70 mediated autophagic degradation of oxidized PRL2 is responsible for osteoclastogenesis and inflammatory bone destruction. Cell Death Differ, 30:647-659. 36182990 10.1038/s41418-022-01068-y PMC9984420

[22] Kimball JS, Johnson JP, Carlson DA (2021). Oxidative Stress and Osteoporosis. J Bone Joint Surg Am, 103:1451-1461. 34014853 10.2106/JBJS.20.00989

[23] Ballabio A, Bonifacino JS (2020). Lysosomes as dynamic regulators of cell and organismal homeostasis. Nat Rev Mol Cell Biol, 21:101-118. 31768005 10.1038/s41580-019-0185-4

[24] Wong YC, Kim S, Peng W, Krainc D (2019). Regulation and Function of Mitochondria-Lysosome Membrane Contact Sites in Cellular Homeostasis. Trends Cell Biol, 29:500-513. 30898429 10.1016/j.tcb.2019.02.004 PMC8475646

[25] Ni HM, Williams JA, Ding WX (2015). Mitochondrial dynamics and mitochondrial quality control. Redox Biol, 4:6-13. 25479550 10.1016/j.redox.2014.11.006 PMC4309858

[26] Chan DC (2020). Mitochondrial Dynamics and Its Involvement in Disease. Annu Rev Pathol, 15:235-259. 31585519 10.1146/annurev-pathmechdis-012419-032711

[27] Lin MT, Beal MF (2006). Mitochondrial dysfunction and oxidative stress in neurodegenerative diseases. Nature, 443:787-795. 17051205 10.1038/nature05292

[28] Mishra P, Chan DC (2014). Mitochondrial dynamics and inheritance during cell division, development and disease. Nat Rev Mol Cell Biol, 15:634-646. 25237825 10.1038/nrm3877 PMC4250044

[29] Wang J, Liu X, Qiu Y, Shi Y, Cai J, Wang B, et al. (2018). Cell adhesion-mediated mitochondria transfer contributes to mesenchymal stem cell-induced chemoresistance on T cell acute lymphoblastic leukemia cells. J Hematol Oncol, 11:11. 29357914 10.1186/s13045-018-0554-z PMC5778754

[30] Singh B, Modica-Napolitano JS, Singh KK (2017). Defining the momiome: Promiscuous information transfer by mobile mitochondria and the mitochondrial genome. Semin Cancer Biol, 47:1-17. 28502611 10.1016/j.semcancer.2017.05.004 PMC5681893

[31] Garbincius JF, Elrod JW (2022). Mitochondrial calcium exchange in physiology and disease. Physiol Rev, 102:893-992. 34698550 10.1152/physrev.00041.2020 PMC8816638

[32] Bravo-Sagua R, Parra V, López-Crisosto C, Díaz P, Quest AF, Lavandero S (2017). Calcium Transport and Signaling in Mitochondria. Compr Physiol, 7:623-634. 28333383 10.1002/cphy.c160013

[33] Amorim JA, Coppotelli G, Rolo AP, Palmeira CM, Ross JM, Sinclair DA (2022). Mitochondrial and metabolic dysfunction in ageing and age-related diseases. Nat Rev Endocrinol, 18:243-258. 35145250 10.1038/s41574-021-00626-7 PMC9059418

[34] Huang HM, Chen HL, Gibson GE (2014). Interactions of endoplasmic reticulum and mitochondria Ca(2+) stores with capacitative calcium entry. Metab Brain Dis, 29:1083-1093. 24748364 10.1007/s11011-014-9541-4 PMC4206688

[35] Eaton S (2002). Control of mitochondrial beta-oxidation flux. Prog Lipid Res, 41:197-239. 11814524 10.1016/s0163-7827(01)00024-8

[36] Eisenberg T, Abdellatif M, Schroeder S, Primessnig U, Stekovic S, Pendl T, et al. (2016). Cardioprotection and lifespan extension by the natural polyamine spermidine. Nat Med, 22:1428-1438. 27841876 10.1038/nm.4222 PMC5806691

[37] Yamamori T, Yasui H, Yamazumi M, Wada Y, Nakamura Y, Nakamura H, et al. (2012). Ionizing radiation induces mitochondrial reactive oxygen species production accompanied by upregulation of mitochondrial electron transport chain function and mitochondrial content under control of the cell cycle checkpoint. Free Radic Biol Med, 53:260-270. 22580337 10.1016/j.freeradbiomed.2012.04.033

[38] Zeng Z, Zhou X, Wang Y, Cao H, Guo J, Wang P, et al. (2022). Mitophagy-A New Target of Bone Disease. Biomolecules, 12. 36291629 10.3390/biom12101420 PMC9599755

[39] Hong SE, Lee J, Seo DH, In Lee H, Ri Park D, Lee GR, et al. (2017). Euphorbia factor L1 inhibits osteoclastogenesis by regulating cellular redox status and induces Fas-mediated apoptosis in osteoclast. Free Radic Biol Med, 112:191-199. 28774817 10.1016/j.freeradbiomed.2017.07.030

[40] Paschall HA, Paschall MM (1975). Electron microscopic observations of 20 human osteosarcomas. Clin Orthop Relat Res:42-56. 10.1097/00003086-197509000-00006 169000

[41] Park KR, Park JI, Lee S, Yoo K, Kweon GR, Kwon IK, et al. (2022). Chi3L1 is a therapeutic target in bone metabolism and a potential clinical marker in patients with osteoporosis. Pharmacol Res, 184:106423. 36064078 10.1016/j.phrs.2022.106423

[42] Wang DK, Zheng HL, Zhou WS, Duan ZW, Jiang SD, Li B, et al. (2022). Mitochondrial Dysfunction in Oxidative Stress-Mediated Intervertebral Disc Degeneration. Orthop Surg, 14:1569-1582. 35673928 10.1111/os.13302 PMC9363752

[43] Miwa S, Kashyap S, Chini E, von Zglinicki T (2022). Mitochondrial dysfunction in cell senescence and aging. J Clin Invest, 132. 10.1172/JCI158447 PMC9246372 35775483

[44] Zhao M, Wang Y, Li L, Liu S, Wang C, Yuan Y, et al. (2021). Mitochondrial ROS promote mitochondrial dysfunction and inflammation in ischemic acute kidney injury by disrupting TFAM-mediated mtDNA maintenance. Theranostics, 11:1845-1863. 33408785 10.7150/thno.50905 PMC7778599

[45] Picca A, Calvani R, Coelho-Junior HJ, Marzetti E (2021). Cell Death and Inflammation: The Role of Mitochondria in Health and Disease. Cells, 10. 33802550 10.3390/cells10030537 PMC7998762

[46] Luo Y, Ma J, Lu W (2020). The Significance of Mitochondrial Dysfunction in Cancer. Int J Mol Sci, 21. 32764295 10.3390/ijms21165598 PMC7460667

[47] Woodburn SC, Bollinger JL, Wohleb ES (2021). The semantics of microglia activation: neuroinflammation, homeostasis, and stress. J Neuroinflammation, 18:258. 34742308 10.1186/s12974-021-02309-6 PMC8571840

[48] Fernie AR, Carrari F, Sweetlove LJ (2004). Respiratory metabolism: glycolysis, the TCA cycle and mitochondrial electron transport. Curr Opin Plant Biol, 7:254-261. 15134745 10.1016/j.pbi.2004.03.007

[49] Rich PR, Maréchal A (2010). The mitochondrial respiratory chain. Essays Biochem, 47:1-23. 20533897 10.1042/bse0470001

[50] Wang S, Qiu L, Liu X, Xu G, Siegert M, Lu Q, et al. (2018). Electron transport chains in organohalide-respiring bacteria and bioremediation implications. Biotechnol Adv, 36:1194-1206. 29631017 10.1016/j.biotechadv.2018.03.018

[51] Biasizzo M, Kopitar-Jerala N (2020). Interplay Between NLRP3 Inflammasome and Autophagy. Front Immunol, 11:591803. 33163006 10.3389/fimmu.2020.591803 PMC7583715

[52] Meyer JN, Leuthner TC, Luz AL (2017). Mitochondrial fusion, fission, and mitochondrial toxicity. Toxicology, 391:42-53. 28789970 10.1016/j.tox.2017.07.019 PMC5681418

[53] Nowinski SM, Solmonson A, Rusin SF, Maschek JA, Bensard CL, Fogarty S, et al. (2020). Mitochondrial fatty acid synthesis coordinates oxidative metabolism in mammalian mitochondria. Elife, 9. 10.7554/eLife.58041 PMC7470841 32804083

[54] McStay GP (2017). Complex formation and turnover of mitochondrial transporters and ion channels. J Bioenerg Biomembr, 49:101-111. 26810820 10.1007/s10863-016-9648-x

[55] Marzetti E, Calvani R, Cesari M, Buford TW, Lorenzi M, Behnke BJ, et al. (2013). Mitochondrial dysfunction and sarcopenia of aging: from signaling pathways to clinical trials. Int J Biochem Cell Biol, 45:2288-2301. 23845738 10.1016/j.biocel.2013.06.024 PMC3759621

[56] Paradies G, Paradies V, Ruggiero FM, Petrosillo G (2019). Role of Cardiolipin in Mitochondrial Function and Dynamics in Health and Disease: Molecular and Pharmacological Aspects. Cells, 8. 31315173 10.3390/cells8070728 PMC6678812

[57] Sun K, Jing X, Guo J, Yao X, Guo F (2021). Mitophagy in degenerative joint diseases. Autophagy, 17:2082-2092. 32967533 10.1080/15548627.2020.1822097 PMC8496714

[58] Oldenburg DJ, Bendich AJ (2015). DNA maintenance in plastids and mitochondria of plants. Front Plant Sci, 6:883. 26579143 10.3389/fpls.2015.00883 PMC4624840

[59] Komori T (2006). Regulation of osteoblast differentiation by transcription factors. J Cell Biochem, 99:1233-1239. 16795049 10.1002/jcb.20958

[60] Boyce BF (2013). Advances in the regulation of osteoclasts and osteoclast functions. J Dent Res, 92:860-867. 23906603 10.1177/0022034513500306 PMC3775372

[61] Dallas SL, Prideaux M, Bonewald LF (2013). The osteocyte: an endocrine cell .. and more. Endocr Rev, 34:658-690. 23612223 10.1210/er.2012-1026 PMC3785641

[62] Boyce RW, Weisbrode SE, Kindig O (1985). Ultrastructural development of hyperosteoidosis in 1,25(OH)2D3-treated rats fed high levels of dietary calcium. Bone, 6:165-172. 3839678 10.1016/8756-3282(85)90049-3

[63] Steiner GC (1977). Ultrastructure of osteoblastoma. Cancer, 39:2127-2136. 265750 10.1002/1097-0142(197705)39:5<2127::aid-cncr2820390529>3.0.co;2-r

[64] Tsupykov O, Ustymenko A, Kyryk V, Smozhanik E, Yatsenko K, Butenko G, et al. (2016). Ultrastructural study of mouse adipose-derived stromal cells induced towards osteogenic direction. Microsc Res Tech, 79:557-564. 27087359 10.1002/jemt.22670

[65] Weisbrode SE, Capen CC, Nagode LA (1974). Effects of parathyroid hormone on bone of thyroparathyroidectomized rats: an ultrastructural and enzymatic study. Am J Pathol, 75:529-541. 4275712 PMC1910842

[66] Stone JA, McCrea JB, Witter R, Zajic S, Stoch SA (2019). Clinical and translational pharmacology of the cathepsin K inhibitor odanacatib studied for osteoporosis. Br J Clin Pharmacol, 85:1072-1083. 30663085 10.1111/bcp.13869 PMC6533439

[67] Prince RL, Glendenning P (2004). 8: Disorders of bone and mineral other than osteoporosis. Med J Aust, 180:354-359. 15059059 10.5694/j.1326-5377.2004.tb05977.x

[68] Park-Min KH (2019). Metabolic reprogramming in osteoclasts. Semin Immunopathol, 41:565-572. 31552471 10.1007/s00281-019-00757-0 PMC7671717

[69] Das BK, Wang L, Fujiwara T, Zhou J, Aykin-Burns N, Krager KJ, et al. (2022). Transferrin receptor 1-mediated iron uptake regulates bone mass in mice via osteoclast mitochondria and cytoskeleton. Elife, 11. 10.7554/eLife.73539 PMC9352353 35758636

[70] Lemma S, Sboarina M, Porporato PE, Zini N, Sonveaux P, Di Pompo G, et al. (2016). Energy metabolism in osteoclast formation and activity. Int J Biochem Cell Biol, 79:168-180. 27590854 10.1016/j.biocel.2016.08.034

[71] Schreurs AS, Torres S, Truong T, Moyer EL, Kumar A, Tahimic CGT, et al. (2020). Skeletal tissue regulation by catalase overexpression in mitochondria. Am J Physiol Cell Physiol, 319:C734-c745. 32783660 10.1152/ajpcell.00068.2020

[72] Cui J, Shibata Y, Zhu T, Zhou J, Zhang J (2022). Osteocytes in bone aging: Advances, challenges, and future perspectives. Ageing Res Rev, 77:101608. 35283289 10.1016/j.arr.2022.101608

[73] Feng M, Zhang R, Gong F, Yang P, Fan L, Ni J, et al. (2014). Protective effects of necrostatin-1 on glucocorticoid-induced osteoporosis in rats. J Steroid Biochem Mol Biol, 144 Pt B:455-462. 25220755 10.1016/j.jsbmb.2014.09.005

[74] Gao J, Qin A, Liu D, Ruan R, Wang Q, Yuan J, et al. (2019). Endoplasmic reticulum mediates mitochondrial transfer within the osteocyte dendritic network. Sci Adv, 5:eaaw7215. 31799389 10.1126/sciadv.aaw7215 PMC6867880

[75] Zhang J, Riquelme MA, Hua R, Acosta FM, Gu S, Jiang JX (2022). Connexin 43 hemichannels regulate mitochondrial ATP generation, mobilization, and mitochondrial homeostasis against oxidative stress. Elife, 11. 10.7554/eLife.82206 PMC9642995 36346745

[76] Hwang HS, Kim HA (2015). Chondrocyte Apoptosis in the Pathogenesis of Osteoarthritis. Int J Mol Sci, 16:26035-26054. 26528972 10.3390/ijms161125943 PMC4661802

[77] Jiang W, Liu H, Wan R, Wu Y, Shi Z, Huang W (2021). Mechanisms linking mitochondrial mechanotransduction and chondrocyte biology in the pathogenesis of osteoarthritis. Ageing Res Rev, 67:101315. 33684550 10.1016/j.arr.2021.101315

[78] Bolduc JA, Collins JA, Loeser RF (2019). Reactive oxygen species, aging and articular cartilage homeostasis. Free Radic Biol Med, 132:73-82. 30176344 10.1016/j.freeradbiomed.2018.08.038 PMC6342625

[79] Geng Y, Hansson GK, Holme E (1992). Interferon-gamma and tumor necrosis factor synergize to induce nitric oxide production and inhibit mitochondrial respiration in vascular smooth muscle cells. Circ Res, 71:1268-1276. 1394884 10.1161/01.res.71.5.1268

[80] Pons R, De Vivo DC (2001). Mitochondrial Disease. Curr Treat Options Neurol, 3:271-288. 11282042 10.1007/s11940-001-0008-7

[81] Vercellino I, Sazanov LA (2022). The assembly, regulation and function of the mitochondrial respiratory chain. Nat Rev Mol Cell Biol, 23:141-161. 34621061 10.1038/s41580-021-00415-0

[82] Barros MH, McStay GP (2020). Modular biogenesis of mitochondrial respiratory complexes. Mitochondrion, 50:94-114. 31669617 10.1016/j.mito.2019.10.008

[83] Dobson PF, Rocha MC, Grady JP, Chrysostomou A, Hipps D, Watson S, et al. (2016). Unique quadruple immunofluorescence assay demonstrates mitochondrial respiratory chain dysfunction in osteoblasts of aged and PolgA(-/-) mice. Sci Rep, 6:31907. 27553587 10.1038/srep31907 PMC4995399

[84] Jewell BE, Xu A, Zhu D, Huang MF, Lu L, Liu M, et al. (2021). Patient-derived iPSCs link elevated mitochondrial respiratory complex I function to osteosarcoma in Rothmund-Thomson syndrome. PLoS Genet, 17:e1009971. 34965247 10.1371/journal.pgen.1009971 PMC8716051

[85] Dobson PF, Dennis EP, Hipps D, Reeve A, Laude A, Bradshaw C, et al. (2020). Mitochondrial dysfunction impairs osteogenesis, increases osteoclast activity, and accelerates age related bone loss. Sci Rep, 10:11643. 32669663 10.1038/s41598-020-68566-2 PMC7363892

[86] King MS, Sharpley MS, Hirst J (2009). Reduction of hydrophilic ubiquinones by the flavin in mitochondrial NADH:ubiquinone oxidoreductase (Complex I) and production of reactive oxygen species. Biochemistry, 48:2053-2062. 19220002 10.1021/bi802282h PMC2651670

[87] Ohnishi T, Ohnishi ST, Shinzawa-Ito K, Yoshikawa S (2008). Functional role of coenzyme Q in the energy coupling of NADH-CoQ oxidoreductase (Complex I): stabilization of the semiquinone state with the application of inside-positive membrane potential to proteoliposomes. Biofactors, 32:13-22. 19096096 10.1002/biof.5520320103 PMC2683760

[88] Hipps D, Dobson PF, Warren C, McDonald D, Fuller A, Filby A, et al. (2022). Detecting respiratory chain defects in osteoblasts from osteoarthritic patients using imaging mass cytometry. Bone, 158:116371. 35192969 10.1016/j.bone.2022.116371

[89] Kampjut D, Sazanov LA (2020). The coupling mechanism of mammalian respiratory complex I. Science, 370. 32972993 10.1126/science.abc4209

[90] Ai Q, Jing Y, Jiang R, Lin L, Dai J, Che Q, et al. (2014). Rotenone, a mitochondrial respiratory complex I inhibitor, ameliorates lipopolysaccharide/D-galactosamine-induced fulminant hepatitis in mice. Int Immunopharmacol, 21:200-207. 24830863 10.1016/j.intimp.2014.04.028

[91] Giorgio V, Schiavone M, Galber C, Carini M, Da Ros T, Petronilli V, et al. (2018). The idebenone metabolite QS10 restores electron transfer in complex I and coenzyme Q defects. Biochim Biophys Acta Bioenerg, 1859:901-908. 29694828 10.1016/j.bbabio.2018.04.006

[92] Pal S, Porwal K, Rajak S, Sinha RA, Chattopadhyay N (2020). Selective dietary polyphenols induce differentiation of human osteoblasts by adiponectin receptor 1-mediated reprogramming of mitochondrial energy metabolism. Biomed Pharmacother, 127:110207. 32422565 10.1016/j.biopha.2020.110207

[93] Porwal K, Pal S, Dev K, China SP, Kumar Y, Singh C, et al. (2017). Guava fruit extract and its triterpene constituents have osteoanabolic effect: Stimulation of osteoblast differentiation by activation of mitochondrial respiration via the Wnt/β-catenin signaling. J Nutr Biochem, 44:22-34. 28343085 10.1016/j.jnutbio.2017.02.011

[94] Lee SY, Long F (2018). Notch signaling suppresses glucose metabolism in mesenchymal progenitors to restrict osteoblast differentiation. J Clin Invest, 128:5573-5586. 30284985 10.1172/JCI96221 PMC6264656

[95] Da W, Tao L, Zhu Y (2021). The Role of Osteoclast Energy Metabolism in the Occurrence and Development of Osteoporosis. Front Endocrinol (Lausanne), 12:675385. 34054735 10.3389/fendo.2021.675385 PMC8150001

[96] Morten KJ, Badder L, Knowles HJ (2013). Differential regulation of HIF-mediated pathways increases mitochondrial metabolism and ATP production in hypoxic osteoclasts. J Pathol, 229:755-764. 23303559 10.1002/path.4159 PMC3618370

[97] Kim HN, Ponte F, Nookaew I, Ucer Ozgurel S, Marques-Carvalho A, Iyer S, et al. (2020). Estrogens decrease osteoclast number by attenuating mitochondria oxidative phosphorylation and ATP production in early osteoclast precursors. Sci Rep, 10:11933. 32686739 10.1038/s41598-020-68890-7 PMC7371870

[98] Kwak HB, Lee BK, Oh J, Yeon JT, Choi SW, Cho HJ, et al. (2010). Inhibition of osteoclast differentiation and bone resorption by rotenone, through down-regulation of RANKL-induced c-Fos and NFATc1 expression. Bone, 46:724-731. 19900598 10.1016/j.bone.2009.10.042

[99] Jin Z, Wei W, Yang M, Du Y, Wan Y (2014). Mitochondrial complex I activity suppresses inflammation and enhances bone resorption by shifting macrophage-osteoclast polarization. Cell Metab, 20:483-498. 25130399 10.1016/j.cmet.2014.07.011 PMC4156549

[100] Hlavácek M (1999). Lubrication of the human ankle joint in walking with the synovial fluid filtrated by the cartilage with the surface zone worn out: steady pure sliding motion. J Biomech, 32:1059-1069. 10476844 10.1016/s0021-9290(99)00095-0

[101] Alvarez-Garcia O, Matsuzaki T, Olmer M, Plate L, Kelly JW, Lotz MK (2017). Regulated in Development and DNA Damage Response 1 Deficiency Impairs Autophagy and Mitochondrial Biogenesis in Articular Cartilage and Increases the Severity of Experimental Osteoarthritis. Arthritis Rheumatol, 69:1418-1428. 28334504 10.1002/art.40104 PMC5489357

[102] López-Armada MJ, Caramés B, Martín MA, Cillero-Pastor B, Lires-Dean M, Fuentes-Boquete I, et al. (2006). Mitochondrial activity is modulated by TNFalpha and IL-1beta in normal human chondrocyte cells. Osteoarthritis Cartilage, 14:1011-1022. 16679036 10.1016/j.joca.2006.03.008

[103] Wang Y, Zhao X, Lotz M, Terkeltaub R, Liu-Bryan R (2015). Mitochondrial biogenesis is impaired in osteoarthritis chondrocytes but reversible via peroxisome proliferator-activated receptor γ coactivator 1α. Arthritis Rheumatol, 67:2141-2153. 25940958 10.1002/art.39182 PMC4519411

[104] Huang LW, Huang TC, Hu YC, Hsieh BS, Chiu PR, Cheng HL, et al. (2020). Zinc protects chondrocytes from monosodium iodoacetate-induced damage by enhancing ATP and mitophagy. Biochem Biophys Res Commun, 521:50-56. 31610916 10.1016/j.bbrc.2019.10.066

[105] Hadrava Vanova K, Kraus M, Neuzil J, Rohlena J (2020). Mitochondrial complex II and reactive oxygen species in disease and therapy. Redox Rep, 25:26-32. 32290794 10.1080/13510002.2020.1752002 PMC7178880

[106] Zhou Q, Zhai Y, Lou J, Liu M, Pang X, Sun F (2011). Thiabendazole inhibits ubiquinone reduction activity of mitochondrial respiratory complex II via a water molecule mediated binding feature. Protein Cell, 2:531-542. 21822798 10.1007/s13238-011-1079-1 PMC4875242

[107] Li Y, Fu G, Gong Y, Li B, Li W, Liu D, et al. (2022). BMP-2 promotes osteogenic differentiation of mesenchymal stem cells by enhancing mitochondrial activity. J Musculoskelet Neuronal Interact, 22:123-131. 35234167 PMC8919656

[108] Ding Y, Yang H, Wang Y, Chen J, Ji Z, Sun H (2017). Sirtuin 3 is required for osteogenic differentiation through maintenance of PGC-1ɑ-SOD2-mediated regulation of mitochondrial function. Int J Biol Sci, 13:254-264. 28255277 10.7150/ijbs.17053 PMC5332879

[109] Michaletti A, Gioia M, Tarantino U, Zolla L (2017). Effects of microgravity on osteoblast mitochondria: a proteomic and metabolomics profile. Sci Rep, 7:15376. 29133864 10.1038/s41598-017-15612-1 PMC5684136

[110] Blanco FJ, López-Armada MJ, Maneiro E (2004). Mitochondrial dysfunction in osteoarthritis. Mitochondrion, 4:715-728. 16120427 10.1016/j.mito.2004.07.022

[111] Blanco FJ, Rego I, Ruiz-Romero C (2011). The role of mitochondria in osteoarthritis. Nat Rev Rheumatol, 7:161-169. 21200395 10.1038/nrrheum.2010.213

[112] Liu JT, Guo X, Ma WJ, Zhang YG, Xu P, Yao JF, et al. (2010). Mitochondrial function is altered in articular chondrocytes of an endemic osteoarthritis, Kashin-Beck disease. Osteoarthritis Cartilage, 18:1218-1226. 20650322 10.1016/j.joca.2010.07.003

[113] Yin M, O'Neill LAJ (2021). The role of the electron transport chain in immunity. Faseb j, 35:e21974. 34793601 10.1096/fj.202101161R

[114] Li G, Wang M, Hao L, Loo WT, Jin L, Cheung MN, et al. (2014). Angiotensin II induces mitochondrial dysfunction and promotes apoptosis via JNK signalling pathway in primary mouse calvaria osteoblast. Arch Oral Biol, 59:513-523. 24632094 10.1016/j.archoralbio.2014.02.015

[115] Choi EM (2011). Glabridin protects osteoblastic MC3T3-E1 cells against antimycin A induced cytotoxicity. Chem Biol Interact, 193:71-78. 21621525 10.1016/j.cbi.2011.05.007

[116] Choi EM, Lee YS (2013). Paeoniflorin isolated from Paeonia lactiflora attenuates osteoblast cytotoxicity induced by antimycin A. Food Funct, 4:1332-1338. 23824342 10.1039/c3fo60147a

[117] Suh KS, Rhee SY, Kim YS, Choi EM (2014). Protective effect of liquiritigenin against methylglyoxal cytotoxicity in osteoblastic MC3T3-E1 cells. Food Funct, 5:1432-1440. 24789098 10.1039/c4fo00127c

[118] Choi EM (2011). Honokiol protects osteoblastic MC3T3-E1 cells against antimycin A-induced cytotoxicity. Inflamm Res, 60:1005-1012. 21800176 10.1007/s00011-011-0360-3

[119] Lee YS, Choi EM (2011). Apocynin stimulates osteoblast differentiation and inhibits bone-resorbing mediators in MC3T3-E1 cells. Cell Immunol, 270:224-229. 21683946 10.1016/j.cellimm.2011.05.011

[120] Liu J, Wang L, Guo X, Pang Q, Wu S, Wu C, et al. (2014). The role of mitochondria in T-2 toxin-induced human chondrocytes apoptosis. PLoS One, 9:e108394. 25264878 10.1371/journal.pone.0108394 PMC4181319

[121] Zhang FJ, Luo W, Lei GH (2015). Role of HIF-1α and HIF-2α in osteoarthritis. Joint Bone Spine, 82:144-147. 25553838 10.1016/j.jbspin.2014.10.003

[122] Gibson JS, Milner PI, White R, Fairfax TP, Wilkins RJ (2008). Oxygen and reactive oxygen species in articular cartilage: modulators of ionic homeostasis. Pflugers Arch, 455:563-573. 17849146 10.1007/s00424-007-0310-7

[123] Cillero-Pastor B, Caramés B, Lires-Deán M, Vaamonde-García C, Blanco FJ, López-Armada MJ (2008). Mitochondrial dysfunction activates cyclooxygenase 2 expression in cultured normal human chondrocytes. Arthritis Rheum, 58:2409-2419. 18668543 10.1002/art.23644

[124] Cillero-Pastor B, Rego-Pérez I, Oreiro N, Fernandez-Lopez C, Blanco FJ (2013). Mitochondrial respiratory chain dysfunction modulates metalloproteases -1, -3 and -13 in human normal chondrocytes in culture. BMC Musculoskelet Disord, 14:235. 23937653 10.1186/1471-2474-14-235 PMC3750811

[125] Yu L, Lin Z, Cheng X, Chu J, Li X, Chen C, et al. (2022). Thorium inhibits human respiratory chain complex IV (cytochrome c oxidase). J Hazard Mater, 424:127546. 34879532 10.1016/j.jhazmat.2021.127546

[126] Gnaiger E, Lassnig B, Kuznetsov A, Rieger G, Margreiter R (1998). Mitochondrial oxygen affinity, respiratory flux control and excess capacity of cytochrome c oxidase. J Exp Biol, 201:1129-1139. 9510525 10.1242/jeb.201.8.1129

[127] Yeh PS, Chen JT, Cherng YG, Yang ST, Tai YT, Chen RM (2020). Methylpiperidinopyrazole Attenuates Estrogen-Induced Mitochondrial Energy Production and Subsequent Osteoblast Maturation via an Estrogen Receptor Alpha-Dependent Mechanism. Molecules, 25. 32580515 10.3390/molecules25122876 PMC7356510

[128] Abramson SB (2008). Osteoarthritis and nitric oxide. Osteoarthritis Cartilage, 16 Suppl 2:S15-20. 18794013 10.1016/S1063-4584(08)60008-4

[129] Jiang H, Ji P, Shang X, Zhou Y (2023). Connection between Osteoarthritis and Nitric Oxide: From Pathophysiology to Therapeutic Target. Molecules, 28. 36838671 10.3390/molecules28041683 PMC9959782

[130] de Andrés MC, Maneiro E, Martín MA, Arenas J, Blanco FJ (2013). Nitric oxide compounds have different effects profiles on human articular chondrocyte metabolism. Arthritis Res Ther, 15:R115. 24025112 10.1186/ar4295 PMC3978712

[131] Holzer T, Probst K, Etich J, Auler M, Georgieva VS, Bluhm B, et al. (2019). Respiratory chain inactivation links cartilage-mediated growth retardation to mitochondrial diseases. J Cell Biol, 218:1853-1870. 31085560 10.1083/jcb.201809056 PMC6548139

[132] Zhang K, Jiang D (2017). RhoA inhibits the hypoxia-induced apoptosis and mitochondrial dysfunction in chondrocytes via positively regulating the CREB phosphorylation. Biosci Rep, 37. 10.1042/BSR20160622 PMC5398256 28254846

[133] Zhang Y, Liu Y, Hou M, Xia X, Liu J, Xu Y, et al. (2023). Reprogramming of Mitochondrial Respiratory Chain Complex by Targeting SIRT3-COX4I2 Axis Attenuates Osteoarthritis Progression. Adv Sci (Weinh), 10:e2206144. 36683245 10.1002/advs.202206144 PMC10074136

[134] Gahura O, Hierro-Yap C, Zíková A (2021). Redesigned and reversed: architectural and functional oddities of the trypanosomal ATP synthase. Parasitology, 148:1151-1160. 33551002 10.1017/S0031182021000202 PMC8311965

[135] Vinogradov AD (2000). Steady-state and pre-steady-state kinetics of the mitochondrial F(1)F(o) ATPase: is ATP synthase a reversible molecular machine? J Exp Biol, 203:41-49. 10600672 10.1242/jeb.203.1.41

[136] Galante YM, Wong SY, Hatefi Y (1981). Mitochondrial adenosinetriphosphatase inhibitor protein: reversible interaction with complex V (ATP synthetase complex). Biochemistry, 20:2671-2678. 6263316 10.1021/bi00512a048

[137] Wyatt CN, Buckler KJ (2004). The effect of mitochondrial inhibitors on membrane currents in isolated neonatal rat carotid body type I cells. J Physiol, 556:175-191. 14724184 10.1113/jphysiol.2003.058131 PMC1664886

[138] Papa S, Zanotti F, Gaballo A (2000). The structural and functional connection between the catalytic and proton translocating sectors of the mitochondrial F1F0-ATP synthase. J Bioenerg Biomembr, 32:401-411. 11768302 10.1023/a:1005584221456

[139] Chuang SC, Liao HJ, Li CJ, Wang GJ, Chang JK, Ho ML (2013). Simvastatin enhances human osteoblast proliferation involved in mitochondrial energy generation. Eur J Pharmacol, 714:74-82. 23769741 10.1016/j.ejphar.2013.05.044

[140] Jorgensen C, Khoury M (2021). Musculoskeletal Progenitor/Stromal Cell-Derived Mitochondria Modulate Cell Differentiation and Therapeutical Function. Front Immunol, 12:606781. 33763061 10.3389/fimmu.2021.606781 PMC7982675

[141] Väänänen HK, Karhukorpi EK, Sundquist K, Wallmark B, Roininen I, Hentunen T, et al. (1990). Evidence for the presence of a proton pump of the vacuolar H(+)-ATPase type in the ruffled borders of osteoclasts. J Cell Biol, 111:1305-1311. 2144003 10.1083/jcb.111.3.1305 PMC2116263

[142] López de Figueroa P, Lotz MK, Blanco FJ, Caramés B (2015). Autophagy activation and protection from mitochondrial dysfunction in human chondrocytes. Arthritis Rheumatol, 67:966-976. 25605458 10.1002/art.39025 PMC4380780

[143] DelCarlo M, Loeser RF (2006). Chondrocyte cell death mediated by reactive oxygen species-dependent activation of PKC-betaI. Am J Physiol Cell Physiol, 290:C802-811. 16236825 10.1152/ajpcell.00214.2005 PMC1482466

[144] Long F (2018). Energy Metabolism and Bone. Bone, 115:1. 30146067 10.1016/j.bone.2018.08.002

[145] Luo B, Zhou X, Tang Q, Yin Y, Feng G, Li S, et al. (2021). Circadian rhythms affect bone reconstruction by regulating bone energy metabolism. J Transl Med, 19:410. 34579752 10.1186/s12967-021-03068-x PMC8477514

[146] Datta HK, Ng WF, Walker JA, Tuck SP, Varanasi SS (2008). The cell biology of bone metabolism. J Clin Pathol, 61:577-587. 18441154 10.1136/jcp.2007.048868

[147] Lee WC, Guntur AR, Long F, Rosen CJ (2017). Energy Metabolism of the Osteoblast: Implications for Osteoporosis. Endocr Rev, 38:255-266. 28472361 10.1210/er.2017-00064 PMC5460680

[148] Weivoda MM, Chew CK, Monroe DG, Farr JN, Atkinson EJ, Geske JR, et al. (2020). Identification of osteoclast-osteoblast coupling factors in humans reveals links between bone and energy metabolism. Nat Commun, 11:87. 31911667 10.1038/s41467-019-14003-6 PMC6946812

[149] Dirckx N, Moorer MC, Clemens TL, Riddle RC (2019). The role of osteoblasts in energy homeostasis. Nat Rev Endocrinol, 15:651-665. 31462768 10.1038/s41574-019-0246-y PMC6958555

[150] Donat A, Knapstein PR, Jiang S, Baranowsky A, Ballhause TM, Frosch KH, et al. (2021). Glucose Metabolism in Osteoblasts in Healthy and Pathophysiological Conditions. Int J Mol Sci, 22. 33923498 10.3390/ijms22084120 PMC8073638

[151] Chen CT, Shih YR, Kuo TK, Lee OK, Wei YH (2008). Coordinated changes of mitochondrial biogenesis and antioxidant enzymes during osteogenic differentiation of human mesenchymal stem cells. Stem Cells, 26:960-968. 18218821 10.1634/stemcells.2007-0509

[152] Li B, Lee WC, Song C, Ye L, Abel ED, Long F (2020). Both aerobic glycolysis and mitochondrial respiration are required for osteoclast differentiation. Faseb j, 34:11058-11067. 32627870 10.1096/fj.202000771R

[153] Miyazaki T, Iwasawa M, Nakashima T, Mori S, Shigemoto K, Nakamura H, et al. (2012). Intracellular and extracellular ATP coordinately regulate the inverse correlation between osteoclast survival and bone resorption. J Biol Chem, 287:37808-37823. 22988253 10.1074/jbc.M112.385369 PMC3488055

[154] Sakakura Y, Shibui T, Irie K, Yajima T (2008). Metabolic mode peculiar to Meckel's cartilage: immunohistochemical comparisons of hypoxia-inducible factor-1alpha and glucose transporters in developing endochondral bones in mice. Eur J Oral Sci, 116:341-352. 18705802 10.1111/j.1600-0722.2008.00548.x

[155] Bae S, Lee MJ, Mun SH, Giannopoulou EG, Yong-Gonzalez V, Cross JR, et al. (2017). MYC-dependent oxidative metabolism regulates osteoclastogenesis via nuclear receptor ERRα. J Clin Invest, 127:2555-2568. 28530645 10.1172/JCI89935 PMC5490751

[156] Stefanovic-Racic M, Stadler J, Georgescu HI, Evans CH (1994). Nitric oxide and energy production in articular chondrocytes. J Cell Physiol, 159:274-280. 8163567 10.1002/jcp.1041590211

[157] Tchetina EV, Markova GA (2018). Regulation of energy metabolism in the growth plate and osteoarthritic chondrocytes. Rheumatol Int, 38:1963-1974. 30019225 10.1007/s00296-018-4103-4

[158] Zhang B, Pan C, Feng C, Yan C, Yu Y, Chen Z, et al. (2022). Role of mitochondrial reactive oxygen species in homeostasis regulation. Redox Rep, 27:45-52. 35213291 10.1080/13510002.2022.2046423 PMC8890532

[159] Antonucci S, Di Lisa F, Kaludercic N (2021). Mitochondrial reactive oxygen species in physiology and disease. Cell Calcium, 94:102344. 33556741 10.1016/j.ceca.2020.102344

[160] Liochev SI (2013). Reactive oxygen species and the free radical theory of aging. Free Radic Biol Med, 60:1-4. 23434764 10.1016/j.freeradbiomed.2013.02.011

[161] Pisoschi AM, Pop A (2015). The role of antioxidants in the chemistry of oxidative stress: A review. Eur J Med Chem, 97:55-74. 25942353 10.1016/j.ejmech.2015.04.040

[162] Le Moal E, Pialoux V, Juban G, Groussard C, Zouhal H, Chazaud B, et al. (2017). Redox Control of Skeletal Muscle Regeneration. Antioxid Redox Signal, 27:276-310. 28027662 10.1089/ars.2016.6782 PMC5685069

[163] Brookes PS (2005). Mitochondrial H(+) leak and ROS generation: an odd couple. Free Radic Biol Med, 38:12-23. 15589367 10.1016/j.freeradbiomed.2004.10.016

[164] Black HS (2022). A Synopsis of the Associations of Oxidative Stress, ROS, and Antioxidants with Diabetes Mellitus. Antioxidants (Basel), 11. 36290725 10.3390/antiox11102003 PMC9598123

[165] Kontos HA (1989). Oxygen radicals in CNS damage. Chem Biol Interact, 72:229-255. 2557981 10.1016/0009-2797(89)90001-x

[166] Islam MT (2017). Oxidative stress and mitochondrial dysfunction-linked neurodegenerative disorders. Neurol Res, 39:73-82. 27809706 10.1080/01616412.2016.1251711

[167] Pigeolet E, Corbisier P, Houbion A, Lambert D, Michiels C, Raes M, et al. (1990). Glutathione peroxidase, superoxide dismutase, and catalase inactivation by peroxides and oxygen derived free radicals. Mech Ageing Dev, 51:283-297. 2308398 10.1016/0047-6374(90)90078-t

[168] Zhao RZ, Jiang S, Zhang L, Yu ZB (2019). Mitochondrial electron transport chain, ROS generation and uncoupling (Review). Int J Mol Med, 44:3-15. 31115493 10.3892/ijmm.2019.4188 PMC6559295

[169] Gill SS, Tuteja N (2010). Reactive oxygen species and antioxidant machinery in abiotic stress tolerance in crop plants. Plant Physiol Biochem, 48:909-930. 20870416 10.1016/j.plaphy.2010.08.016

[170] Sandhir R, Halder A, Sunkaria A (2017). Mitochondria as a centrally positioned hub in the innate immune response. Biochim Biophys Acta Mol Basis Dis, 1863:1090-1097. 27794419 10.1016/j.bbadis.2016.10.020

[171] Banoth B, Cassel SL (2018). Mitochondria in innate immune signaling. Transl Res, 202:52-68. 30165038 10.1016/j.trsl.2018.07.014 PMC6218307

[172] Rodríguez-Nuevo A, Torres-Sanchez A, Duran JM, De Guirior C, Martínez-Zamora MA, Böke E (2022). Oocytes maintain ROS-free mitochondrial metabolism by suppressing complex I. Nature, 607:756-761. 35859172 10.1038/s41586-022-04979-5 PMC9329100

[173] Zhang Y, Guo L, Han S, Chen L, Li C, Zhang Z, et al. (2020). Adult mesenchymal stem cell ageing interplays with depressed mitochondrial Ndufs6. Cell Death Dis, 11:1075. 33323934 10.1038/s41419-020-03289-w PMC7738680

[174] Melcher M, Danhauser K, Seibt A, Degistirici Ö, Baertling F, Kondadi AK, et al. (2017). Modulation of oxidative phosphorylation and redox homeostasis in mitochondrial NDUFS4 deficiency via mesenchymal stem cells. Stem Cell Res Ther, 8:150. 28646906 10.1186/s13287-017-0601-7 PMC5482938

[175] Zhang J, Hu W, Ding C, Yao G, Zhao H, Wu S (2019). Deferoxamine inhibits iron-uptake stimulated osteoclast differentiation by suppressing electron transport chain and MAPKs signaling. Toxicol Lett, 313:50-59. 31238089 10.1016/j.toxlet.2019.06.007

[176] Murphy MP (2009). How mitochondria produce reactive oxygen species. Biochem J, 417:1-13. 19061483 10.1042/BJ20081386 PMC2605959

[177] Quinlan CL, Orr AL, Perevoshchikova IV, Treberg JR, Ackrell BA, Brand MD (2012). Mitochondrial complex II can generate reactive oxygen species at high rates in both the forward and reverse reactions. J Biol Chem, 287:27255-27264. 22689576 10.1074/jbc.M112.374629 PMC3411067

[178] Wang J, Zhang Y, Cao J, Wang Y, Anwar N, Zhang Z, et al. (2023). The role of autophagy in bone metabolism and clinical significance. Autophagy:1-19. 10.1080/15548627.2023.2186112 PMC10392742 36858962

[179] Cela O, Piccoli C, Scrima R, Quarato G, Marolla A, Cinnella G, et al. (2010). Bupivacaine uncouples the mitochondrial oxidative phosphorylation, inhibits respiratory chain complexes I and III and enhances ROS production: results of a study on cell cultures. Mitochondrion, 10:487-496. 20546950 10.1016/j.mito.2010.05.005

[180] Sánchez-Duarte E, Cortés-Rojo C, Sánchez-Briones LA, Campos-García J, Saavedra-Molina A, Delgado-Enciso I, et al. (2020). Nicorandil Affects Mitochondrial Respiratory Chain Function by Increasing Complex III Activity and ROS Production in Skeletal Muscle Mitochondria. J Membr Biol, 253:309-318. 32620983 10.1007/s00232-020-00129-y

[181] Erecińska M, Wilson DF (1976). The effect of antimycin A on cytochromes b561, b566, and their relationship to ubiquinone and the iron-sulfer centers S-1 (+N-2) and S-3. Arch Biochem Biophys, 174:143-157. 180891 10.1016/0003-9861(76)90333-7

[182] Li S, Kim MJ, Lee SH, Jin L, Cong W, Jeong HG, et al. (2021). Metallothionein 3 Promotes Osteoblast Differentiation in C2C12 Cells via Reduction of Oxidative Stress. Int J Mol Sci, 22. 33919218 10.3390/ijms22094312 PMC8122383

[183] Banerjee R, Purhonen J, Kallijärvi J (2022). The mitochondrial coenzyme Q junction and complex III: biochemistry and pathophysiology. Febs j, 289:6936-6958. 34428349 10.1111/febs.16164

[184] Armstrong JS, Whiteman M, Rose P, Jones DP (2003). The Coenzyme Q10 analog decylubiquinone inhibits the redox-activated mitochondrial permeability transition: role of mitcohondrial [correction mitochondrial] complex III. J Biol Chem, 278:49079-49084. 12949071 10.1074/jbc.M307841200

[185] Zheng D, Cui C, Yu M, Li X, Wang L, Chen X, et al. (2018). Coenzyme Q10 promotes osteoblast proliferation and differentiation and protects against ovariectomy-induced osteoporosis. Mol Med Rep, 17:400-407. 29115467 10.3892/mmr.2017.7907

[186] Moon HJ, Ko WK, Jung MS, Kim JH, Lee WJ, Park KS, et al. (2013). Coenzyme q10 regulates osteoclast and osteoblast differentiation. J Food Sci, 78:H785-891. 23582186 10.1111/1750-3841.12116

[187] Zheng D, Cui C, Shao C, Wang Y, Ye C, Lv G (2021). Coenzyme Q10 inhibits RANKL-induced osteoclastogenesis by regulation of mitochondrial apoptosis and oxidative stress in RAW264.7 cells. J Biochem Mol Toxicol, 35:e22778. 33754447 10.1002/jbt.22778

[188] Zhang XX, Qian KJ, Zhang Y, Wang ZJ, Yu YB, Liu XJ, et al. (2015). Efficacy of coenzyme Q10 in mitigating spinal cord injury-induced osteoporosis. Mol Med Rep, 12:3909-3915. 26016719 10.3892/mmr.2015.3856

[189] Lee J, Hong YS, Jeong JH, Yang EJ, Jhun JY, Park MK, et al. (2013). Coenzyme Q10 ameliorates pain and cartilage degradation in a rat model of osteoarthritis by regulating nitric oxide and inflammatory cytokines. PLoS One, 8:e69362. 23894457 10.1371/journal.pone.0069362 PMC3718733

[190] Deng C, Zhang Q, He P, Zhou B, He K, Sun X, et al. (2021). Targeted apoptosis of macrophages and osteoclasts in arthritic joints is effective against advanced inflammatory arthritis. Nat Commun, 12:2174. 33846342 10.1038/s41467-021-22454-z PMC8042091

[191] Wang YJ, Yan J, Zou XL, Guo KJ, Zhao Y, Meng CY, et al. (2017). Bone marrow mesenchymal stem cells repair cadmium-induced rat testis injury by inhibiting mitochondrial apoptosis. Chem Biol Interact, 271:39-47. 28457857 10.1016/j.cbi.2017.04.024

[192] Abate M, Festa A, Falco M, Lombardi A, Luce A, Grimaldi A, et al. (2020). Mitochondria as playmakers of apoptosis, autophagy and senescence. Semin Cell Dev Biol, 98:139-153. 31154010 10.1016/j.semcdb.2019.05.022

[193] Hock JM, Krishnan V, Onyia JE, Bidwell JP, Milas J, Stanislaus D (2001). Osteoblast apoptosis and bone turnover. J Bone Miner Res, 16:975-984. 11393794 10.1359/jbmr.2001.16.6.975

[194] Komori T (2016). Cell Death in Chondrocytes, Osteoblasts, and Osteocytes. Int J Mol Sci, 17. 27929439 10.3390/ijms17122045 PMC5187845

[195] Fischer V, Haffner-Luntzer M (2022). Interaction between bone and immune cells: Implications for postmenopausal osteoporosis. Semin Cell Dev Biol, 123:14-21. 34024716 10.1016/j.semcdb.2021.05.014

[196] Park DR, Kim J, Kim GM, Lee H, Kim M, Hwang D, et al. (2020). Osteoclast-associated receptor blockade prevents articular cartilage destruction via chondrocyte apoptosis regulation. Nat Commun, 11:4343. 32859940 10.1038/s41467-020-18208-y PMC7455568

[197] Roux S, Lambert-Comeau P, Saint-Pierre C, Lépine M, Sawan B, Parent JL (2005). Death receptors, Fas and TRAIL receptors, are involved in human osteoclast apoptosis. Biochem Biophys Res Commun, 333:42-50. 15936719 10.1016/j.bbrc.2005.05.092

[198] Hosseinzadeh A, Kamrava SK, Joghataei MT, Darabi R, Shakeri-Zadeh A, Shahriari M, et al. (2016). Apoptosis signaling pathways in osteoarthritis and possible protective role of melatonin. J Pineal Res, 61:411-425. 27555371 10.1111/jpi.12362

[199] Moe A, Di Trani J, Rubinstein JL, Brzezinski P (2021). Cryo-EM structure and kinetics reveal electron transfer by 2D diffusion of cytochrome c in the yeast III-IV respiratory supercomplex. Proc Natl Acad Sci U S A, 118. 10.1073/pnas.2021157118 PMC7980474 33836592

[200] Xu F, Ren L, Song M, Shao B, Han Y, Cao Z, et al. (2018). Fas- and Mitochondria-Mediated Signaling Pathway Involved in Osteoblast Apoptosis Induced by AlCl(3). Biol Trace Elem Res, 184:173-185. 29027106 10.1007/s12011-017-1176-y

[201] Zhan J, Yan Z, Zhao M, Qi W, Lin J, Lin Z, et al. (2020). Allicin inhibits osteoblast apoptosis and steroid-induced necrosis of femoral head progression by activating the PI3K/AKT pathway. Food Funct, 11:7830-7841. 32808945 10.1039/d0fo00837k

[202] Yu D, Mu S, Zhao D, Wang G, Chen Z, Ren H, et al. (2015). Puerarin attenuates glucocorticoid-induced apoptosis of hFOB1.19 cells through the JNK- and Akt-mediated mitochondrial apoptotic pathways. Int J Mol Med, 36:345-354. 26101183 10.3892/ijmm.2015.2258 PMC4501663

[203] Gao JM, Li R, Zhang L, Jia LL, Ying XX, Dou DQ, et al. (2013). Cuscuta chinensis seeds water extraction protecting murine osteoblastic MC3T3-E1 cells against tertiary butyl hydroperoxide induced injury. J Ethnopharmacol, 148:587-595. 23702038 10.1016/j.jep.2013.05.005

[204] Angireddy R, Kazmi HR, Srinivasan S, Sun L, Iqbal J, Fuchs SY, et al. (2019). Cytochrome c oxidase dysfunction enhances phagocytic function and osteoclast formation in macrophages. Faseb j, 33:9167-9181. 31063702 10.1096/fj.201900010RR PMC6662975

[205] Ansari MY, Ball HC, Wase SJ, Novak K, Haqqi TM (2021). Lysosomal dysfunction in osteoarthritis and aged cartilage triggers apoptosis in chondrocytes through BAX mediated release of Cytochrome c. Osteoarthritis Cartilage, 29:100-112. 33161099 10.1016/j.joca.2020.08.014 PMC8418332

[206] Zhuang C, Ni S, Yang ZC, Liu RP (2020). Oxidative Stress Induces Chondrocyte Apoptosis through Caspase-Dependent and Caspase-Independent Mitochondrial Pathways and the Antioxidant Mechanism of Angelica Sinensis Polysaccharide. Oxid Med Cell Longev, 2020:3240820. 33224431 10.1155/2020/3240820 PMC7669361

[207] Fleig A, Parekh AB (2017). New insights into Ca(2+) channel function in health and disease. J Physiol, 595:2997-2998. 28503825 10.1113/JP274289 PMC5430230

[208] Publicover SJ, Barratt CL (1999). Voltage-operated Ca2+ channels and the acrosome reaction: which channels are present and what do they do? Hum Reprod, 14:873-879. 10221211 10.1093/humrep/14.4.873

[209] Goltzman D, Hendy GN (2015). The calcium-sensing receptor in bone--mechanistic and therapeutic insights. Nat Rev Endocrinol, 11:298-307. 25752283 10.1038/nrendo.2015.30

[210] Cao C, Ren Y, Barnett AS, Mirando AJ, Rouse D, Mun SH, et al. (2017). Increased Ca2+ signaling through CaV1.2 promotes bone formation and prevents estrogen deficiency-induced bone loss. JCI Insight, 2. 10.1172/jci.insight.95512 PMC5752375 29202453

[211] D'Ambrosio J, Fatatis A (2009). Osteoblasts modulate Ca2+ signaling in bone-metastatic prostate and breast cancer cells. Clin Exp Metastasis, 26:955-964. 19768662 10.1007/s10585-009-9286-3

[212] Sforna L, Megaro A, Pessia M, Franciolini F, Catacuzzeno L (2018). Structure, Gating and Basic Functions of the Ca2+-activated K Channel of Intermediate Conductance. Curr Neuropharmacol, 16:608-617. 28875832 10.2174/1570159X15666170830122402 PMC5997868

[213] Csordás G, Weaver D, Hajnóczky G (2018). Endoplasmic Reticulum-Mitochondrial Contactology: Structure and Signaling Functions. Trends Cell Biol, 28:523-540. 29588129 10.1016/j.tcb.2018.02.009 PMC6005738

[214] Rizzuto R, De Stefani D, Raffaello A, Mammucari C (2012). Mitochondria as sensors and regulators of calcium signalling. Nat Rev Mol Cell Biol, 13:566-578. 22850819 10.1038/nrm3412

[215] Gunter TE, Buntinas L, Sparagna G, Eliseev R, Gunter K (2000). Mitochondrial calcium transport: mechanisms and functions. Cell Calcium, 28:285-296. 11115368 10.1054/ceca.2000.0168

[216] Liu X, Hussain R, Mehmood K, Tang Z, Zhang H, Li Y (2022). Mitochondrial-Endoplasmic Reticulum Communication-Mediated Oxidative Stress and Autophagy. Biomed Res Int, 2022:6459585. 36164446 10.1155/2022/6459585 PMC9509228

[217] Hernansanz-Agustín P, Choya-Foces C, Carregal-Romero S, Ramos E, Oliva T, Villa-Piña T, et al. (2020). Na(+) controls hypoxic signalling by the mitochondrial respiratory chain. Nature, 586:287-291. 32728214 10.1038/s41586-020-2551-y PMC7992277

[218] Haak LL, Grimaldi M, Russell JT (2000). Mitochondria in myelinating cells: calcium signaling in oligodendrocyte precursor cells. Cell Calcium, 28:297-306. 11115369 10.1054/ceca.2000.0176

[219] Foretz M, Guigas B, Bertrand L, Pollak M, Viollet B (2014). Metformin: from mechanisms of action to therapies. Cell Metab, 20:953-966. 25456737 10.1016/j.cmet.2014.09.018

[220] Jiating L, Buyun J, Yinchang Z (2019). Role of Metformin on Osteoblast Differentiation in Type 2 Diabetes. Biomed Res Int, 2019:9203934. 31886264 10.1155/2019/9203934 PMC6899291

[221] Liu L, Li N, Zhao Z, Li W, Xia W (2015). Novel WISP3 mutations causing spondyloepiphyseal dysplasia tarda with progressive arthropathy in two unrelated Chinese families. Joint Bone Spine, 82:125-128. 25553839 10.1016/j.jbspin.2014.10.005

[222] Repudi SR, Patra M, Sen M (2013). WISP3-IGF1 interaction regulates chondrocyte hypertrophy. J Cell Sci, 126:1650-1658. 23424195 10.1242/jcs.119859

[223] Padhan DK, Sengupta A, Patra M, Ganguly A, Mahata SK, Sen M (2020). CCN6 regulates mitochondrial respiratory complex assembly and activity. Faseb j, 34:12163-12176. 32686858 10.1096/fj.202000405RR

[224] Twigg SM (2018). Regulation and bioactivity of the CCN family of genes and proteins in obesity and diabetes. J Cell Commun Signal, 12:359-368. 29411334 10.1007/s12079-018-0458-2 PMC5842210

[225] Brookes PS, Yoon Y, Robotham JL, Anders MW, Sheu SS (2004). Calcium, ATP, and ROS: a mitochondrial love-hate triangle. Am J Physiol Cell Physiol, 287:C817-833. 15355853 10.1152/ajpcell.00139.2004

[226] Mbaya E, Oulès B, Caspersen C, Tacine R, Massinet H, Pennuto M, et al. (2010). Calcium signalling-dependent mitochondrial dysfunction and bioenergetics regulation in respiratory chain Complex II deficiency. Cell Death Differ, 17:1855-1866. 20489732 10.1038/cdd.2010.51

[227] Jünemann S, Heathcote P, Rich PR (1998). On the mechanism of quinol oxidation in the bc1 complex. J Biol Chem, 273:21603-21607. 9705291 10.1074/jbc.273.34.21603

[228] Choi EM (2012). Regulation of intracellular Ca(2+) by reactive oxygen species in osteoblasts treated with antimycin A. J Appl Toxicol, 32:118-125. 21381053 10.1002/jat.1642

[229] Horn TF, Wolf G, Duffy S, Weiss S, Keilhoff G, MacVicar BA (2002). Nitric oxide promotes intracellular calcium release from mitochondria in striatal neurons. Faseb j, 16:1611-1622. 12374784 10.1096/fj.02-0126com

[230] Xu W, Charles IG, Moncada S (2005). Nitric oxide: orchestrating hypoxia regulation through mitochondrial respiration and the endoplasmic reticulum stress response. Cell Res, 15:63-65. 15686630 10.1038/sj.cr.7290267

[231] Bogeski I, Gulaboski R, Kappl R, Mirceski V, Stefova M, Petreska J, et al. (2011). Calcium binding and transport by coenzyme Q. J Am Chem Soc, 133:9293-9303. 21548646 10.1021/ja110190t

[232] Biswas G, Anandatheerthavarada HK, Zaidi M, Avadhani NG (2003). Mitochondria to nucleus stress signaling: a distinctive mechanism of NFkappaB/Rel activation through calcineurin-mediated inactivation of IkappaBbeta. J Cell Biol, 161:507-519. 12732617 10.1083/jcb.200211104 PMC2172940

[233] Biswas G, Adebanjo OA, Freedman BD, Anandatheerthavarada HK, Vijayasarathy C, Zaidi M, et al. (1999). Retrograde Ca2+ signaling in C2C12 skeletal myocytes in response to mitochondrial genetic and metabolic stress: a novel mode of inter-organelle crosstalk. Embo j, 18:522-533. 9927412 10.1093/emboj/18.3.522 PMC1171145

[234] Jiménez-Loygorri JI, Benítez-Fernández R, Viedma-Poyatos Á, Zapata-Muñoz J, Villarejo-Zori B, Gómez-Sintes R, et al. (2023). Mitophagy in the retina: Viewing mitochondrial homeostasis through a new lens. Prog Retin Eye Res, 96:101205. 37454969 10.1016/j.preteyeres.2023.101205

[235] Zhang SM, Fan B, Li YL, Zuo ZY, Li GY (2023). Oxidative Stress-Involved Mitophagy of Retinal Pigment Epithelium and Retinal Degenerative Diseases. Cell Mol Neurobiol. 10.1007/s10571-023-01383-z PMC10477140 37391574

[236] Becker PH, Thérond P, Gaignard P (2023). Targeting mitochondrial function in macrophages: A novel treatment strategy for atherosclerotic cardiovascular disease? Pharmacol Ther, 247:108441. 37201736 10.1016/j.pharmthera.2023.108441

[237] Su L, Zhang J, Gomez H, Kellum JA, Peng Z (2023). Mitochondria ROS and mitophagy in acute kidney injury. Autophagy, 19:401-414. 35678504 10.1080/15548627.2022.2084862 PMC9851232

[238] Lin Q, Li S, Jiang N, Shao X, Zhang M, Jin H, et al. (2019). PINK1-parkin pathway of mitophagy protects against contrast-induced acute kidney injury via decreasing mitochondrial ROS and NLRP3 inflammasome activation. Redox Biol, 26:101254. 31229841 10.1016/j.redox.2019.101254 PMC6597739

[239] Boyman L, Karbowski M, Lederer WJ (2020). Regulation of Mitochondrial ATP Production: Ca(2+) Signaling and Quality Control. Trends Mol Med, 26:21-39. 31767352 10.1016/j.molmed.2019.10.007 PMC7921598

[240] Wang S, Deng Z, Ma Y, Jin J, Qi F, Li S, et al. (2020). The Role of Autophagy and Mitophagy in Bone Metabolic Disorders. Int J Biol Sci, 16:2675-2691. 32792864 10.7150/ijbs.46627 PMC7415419

[241] Li W, Jiang WS, Su YR, Tu KW, Zou L, Liao CR, et al. (2023). PINK1/Parkin-mediated mitophagy inhibits osteoblast apoptosis induced by advanced oxidation protein products. Cell Death Dis, 14:88. 36750550 10.1038/s41419-023-05595-5 PMC9905061

[242] Onishi M, Yamano K, Sato M, Matsuda N, Okamoto K (2021). Molecular mechanisms and physiological functions of mitophagy. Embo j, 40:e104705. 33438778 10.15252/embj.2020104705 PMC7849173

[243] Xu K, Lu C, Ren X, Wang J, Xu P, Zhang Y (2021). Overexpression of HIF-1α enhances the protective effect of mitophagy on steroid-induced osteocytes apoptosis. Environ Toxicol, 36:2123-2137. 34310007 10.1002/tox.23327

[244] Chen L, Shi X, Xie J, Weng SJ, Xie ZJ, Tang JH, et al. (2021). Apelin-13 induces mitophagy in bone marrow mesenchymal stem cells to suppress intracellular oxidative stress and ameliorate osteoporosis by activation of AMPK signaling pathway. Free Radic Biol Med, 163:356-368. 33385540 10.1016/j.freeradbiomed.2020.12.235

[245] Liu P, Cui Y, Liu M, Xiao B, Zhang J, Huang W, et al. (2021). Protective effect of mitophagy against aluminum-induced MC3T3-E1 cells dysfunction. Chemosphere, 282:131086. 34119729 10.1016/j.chemosphere.2021.131086

[246] Huang T, Wang Y, Yu Z, Miao X, Jiang Z, Yu K, et al. (2023). Effect of mitophagy in the formation of osteomorphs derived from osteoclasts. iScience, 26:106682. 37250312 10.1016/j.isci.2023.106682 PMC10214740

[247] Sarkar J, Das M, Howlader MSI, Prateeksha P, Barthels D, Das H (2022). Epigallocatechin-3-gallate inhibits osteoclastic differentiation by modulating mitophagy and mitochondrial functions. Cell Death Dis, 13:908. 36307395 10.1038/s41419-022-05343-1 PMC9616829

[248] Yao H, Xiang L, Huang Y, Tan J, Shen Y, Li F, et al. (2023). Guizhi Shaoyao Zhimu granules attenuate bone destruction in mice with collagen-induced arthritis by promoting mitophagy of osteoclast precursors to inhibit osteoclastogenesis. Phytomedicine, 118:154967. 37490802 10.1016/j.phymed.2023.154967

[249] Zhu L, Wang Z, Sun X, Yu J, Li T, Zhao H, et al. (2023). STAT3/Mitophagy Axis Coordinates Macrophage NLRP3 Inflammasome Activation and Inflammatory Bone Loss. J Bone Miner Res, 38:335-353. 36502520 10.1002/jbmr.4756

[250] Xu K, He Y, Moqbel SAA, Zhou X, Wu L, Bao J (2021). SIRT3 ameliorates osteoarthritis via regulating chondrocyte autophagy and apoptosis through the PI3K/Akt/mTOR pathway. Int J Biol Macromol, 175:351-360. 33556400 10.1016/j.ijbiomac.2021.02.029

[251] Blanco FJ, Rego-Pérez I (2018). Mitochondria and mitophagy: biosensors for cartilage degradation and osteoarthritis. Osteoarthritis Cartilage, 26:989-991. 29857157 10.1016/j.joca.2018.05.018

[252] Jiang N, Xing B, Peng R, Shang J, Wu B, Xiao P, et al. (2022). Inhibition of Cpt1a alleviates oxidative stress-induced chondrocyte senescence via regulating mitochondrial dysfunction and activating mitophagy. Mech Ageing Dev, 205:111688. 35728631 10.1016/j.mad.2022.111688

[253] Xin R, Xu Y, Long D, Mao G, Liao H, Zhang Z, et al. (2022). Mitochonic Acid-5 Inhibits Reactive Oxygen Species Production and Improves Human Chondrocyte Survival by Upregulating SIRT3-Mediated, Parkin-dependent Mitophagy. Front Pharmacol, 13:911716. 35734404 10.3389/fphar.2022.911716 PMC9207248

[254] Jin Z, Chang B, Wei Y, Yang Y, Zhang H, Liu J, et al. (2022). Curcumin exerts chondroprotective effects against osteoarthritis by promoting AMPK/PINK1/Parkin-mediated mitophagy. Biomed Pharmacother, 151:113092. 35550528 10.1016/j.biopha.2022.113092

[255] Hollenberg AM, Huber A, Smith CO, Eliseev RA (2021). Electromagnetic stimulation increases mitochondrial function in osteogenic cells and promotes bone fracture repair. Sci Rep, 11:19114. 34580378 10.1038/s41598-021-98625-1 PMC8476611

[256] Guo Y, Chi X, Wang Y, Heng BC, Wei Y, Zhang X, et al. (2020). Mitochondria transfer enhances proliferation, migration, and osteogenic differentiation of bone marrow mesenchymal stem cell and promotes bone defect healing. Stem Cell Res Ther, 11:245. 32586355 10.1186/s13287-020-01704-9 PMC7318752

[257] Suh J, Kim NK, Shim W, Lee SH, Kim HJ, Moon E, et al. (2023). Mitochondrial fragmentation and donut formation enhance mitochondrial secretion to promote osteogenesis. Cell Metab, 35:345-360.e347. 36754021 10.1016/j.cmet.2023.01.003

[258] Wang X, Shen K, Wang J, Liu K, Wu G, Li Y, et al. (2020). Hypoxic preconditioning combined with curcumin promotes cell survival and mitochondrial quality of bone marrow mesenchymal stem cells, and accelerates cutaneous wound healing via PGC-1α/SIRT3/HIF-1α signaling. Free Radic Biol Med, 159:164-176. 32745765 10.1016/j.freeradbiomed.2020.07.023

[259] Zhou Q, Chen W, Gu C, Liu H, Hu X, Deng L, et al. (2023). Selenium-modified bone cement promotes osteoporotic bone defect repair in ovariectomized rats by restoring GPx1-mediated mitochondrial antioxidant functions. Regen Biomater, 10:rbad011. 36852397 10.1093/rb/rbad011 PMC9960915

[260] Faas MM, de Vos P (2020). Mitochondrial function in immune cells in health and disease. Biochim Biophys Acta Mol Basis Dis, 1866:165845. 32473386 10.1016/j.bbadis.2020.165845

[261] Edwards IR, Bleehen SS (1974). Drug interaction. 3. Br J Dermatol, 90:117-121. 4590662

